# Dual RNA-Seq Reveals Temperature-Mediated Gene Reprogramming and Molecular Crosstalk between Grapevine and *Lasiodiplodia theobromae*

**DOI:** 10.3390/jof9121197

**Published:** 2023-12-14

**Authors:** Junbo Peng, Yonghua Li, Qikai Xing, Caiping Huang, Jiye Yan

**Affiliations:** Beijing Key Laboratory of Environment Friendly Management on Fruit Diseases and Pests in North China, Institute of Plant Protection, Beijing Academy of Agriculture and Forestry Sciences, Beijing 100097, Chinaqikaixing@163.com (Q.X.);

**Keywords:** dual RNA-seq, temperature, transcription reprogramming, grapevine, *Lasiodiplodia theobromae*

## Abstract

High temperatures associated with a fluctuating climate profoundly accelerate the occurrence of a myriad of plant diseases around the world. A comprehensive insight into how plants respond to pathogenic microorganisms under high-temperature stress is required for plant disease management, whereas the underlying mechanisms behind temperature-mediated plant immunity and pathogen pathogenicity are still unclear. Here, we evaluated the effect of high temperature on the development of grapevine canker disease and quantified the contribution of temperature variation to the gene transcription reprogramming of grapevine and its pathogenic agent *Lasiodiplodia theobromae* using a dual RNA-seq approach. The results showed that both grapevine and the pathogen displayed altered transcriptomes under different temperatures, and even the transcription of a plethora of genes from the two organisms responded in different directions and magnitudes. The transcription variability that arose due to temperature oscillation allowed us to identify a total of 26 grapevine gene modules and 17 fungal gene modules that were correlated with more than one gene module of the partner organism, which revealed an extensive web of plant–pathogen gene reprogramming during infection. More importantly, we identified a set of temperature-responsive genes that were transcriptionally orchestrated within the given gene modules. These genes are predicted to be involved in multiple cellular processes including protein folding, stress response regulation, and carbohydrate and peptide metabolisms in grapevine and porphyrin- and pteridine-containing compound metabolisms in *L. theobromae*, implying that in response to temperature oscillation, a complex web of signaling pathways in two organism cells is activated during infection. This study describes a co-transcription network of grapevine and *L. theobromae* in the context of considering temperature variation, which provides novel insights into deciphering the molecular mechanisms underlying temperature-modulated disease development.

## 1. Introduction

The filamentous fungus *Lasiodiplodia theobromae*, a member of the *Botryosphaeriaceae* family, has adapted to infecting over 500 plant species, including grapevine [1], peach [2], cacao [3], mulberry [4], longan [5], and macadamia [6], in the tropics and subtropics [7]. The grapevine canker disease caused by *L. theobromae* has become one of the most notorious trunk diseases and results in considerable damage to the viticulture economy globally. Because of its serious threat to viticulture development, *L. theobromae* has received increasing attention from pathologists and orchardists globally [1]. Over the past decade, a series of research studies on *L. theobromae*, ranging from strain isolation and identification to disease diagnosis and symptom description, epidemiology investigation, and pathogen prevention and control, have already achieved great progress [8,9,10]. Additionally, some publications claim that external stimuli resulting from host behavior changes, microbial equilibrium disruption, and extreme environmental events trigger the fungus to change its lifestyle from endophytic to pathogenic, rendering this organism an opportunistic plant pathogen [11]. However, the molecular mechanism underlying environmental factor-mediated disease occurrence remains to be elucidated.

In the 21st century, extreme weather events, such as heat waves caused by global climate change, are predicted to become more frequent and more intense, thus becoming a formidable threat to the increasing demand for crop production for the growing human population [12,13]. In nature, high temperatures have become major environmental factors that profoundly influence plant growth and development, as well as their interaction with a myriad of pathogenic, commensal, and beneficial microbes [12,14]. As plants and microbes have optimal temperatures for their growth and reproduction, the effect of temperature variables on the mode and outcome of plant–microbe interactions can be favorable, neutral, or adverse [15,16,17]. Generally, the further the temperature deviates from the optimal disease temperature, the fewer disease symptoms will occur in plants [17]. 

In terms of pathosystems, the effects of fluctuating temperatures vary greatly. For example, elevated temperature increased the virulence of the soft-rotting bacterium *Erwinia carotovora* subsp. *carotovora* strain EC153, which was associated with an increased production of extracellular cell-wall-degrading enzymes and the quorum-sensing signal *N*-acyl-homoserine lactone, as well as an accelerated growth rate [12,18]. Conversely, in the case of *Pseudomonas syringae*, the production of the phytotoxin coronatine and the secretion of Type III system proteins HrmA and AvrPto were substantially reduced under a warm temperature compared to a cool temperature [15,19,20]. Similarly, plants experiencing different temperatures also exhibit fundamentally different responses [13]. It has long been known that a high temperature compromises effector-triggered immunity (ETI) in plants; therefore, the elevated temperature has emerged as a great concern for the crops that rely on ETI for disease control [12]. For example, the nucleotide-binding and leucine-rich repeat (NLR) proteins SNC1, together with N and RPS4, do not mount an effective resistance at high temperatures [14,21]. However, mutations in SNC1 and N proteins restored their nuclear accumulation and activities at a high temperature [14]. Additionally, abscisic acid (ABA) deficiency restored immunity activation by enhancing the nuclear accumulation of SNC1 and RPS4 proteins in Arabidopsis at an elevated temperature [21]. These data suggest that increased nuclear localizations of NLR proteins including N, SNC1, and RPS4 likely contribute to temperature-mediated defense responses in plants. However, the nucleo-cytoplasmic partitioning of NLR proteins may not be generalized as a common mechanism for the temperature sensitivity of ETI, as not all NLRs showed nuclear localization, and some NLR proteins even function better in warmer temperatures [12]. For example, the rice gene *Xa7* conferred resistance against the bacterium *Xanthomonas oryzae* pv. *oryzae* (*Xoo*), and inhibited disease more effectively at a high temperature than at a low temperature, which was likely correlated with the suppressed expression of ABA-responsive genes [22,23]. Recently, a growing body of evidence revealed that the effect of elevated temperature on plant immunity surpasses the ETI pathway [12]. For example, the increased susceptibility of Arabidopsis to *Pseudomonas syringae* pv. *tomato* (*Pst*) DC3000 at elevated temperatures was associated with significant reductions in salicylic acid (SA) biosynthesis and SA-related defense gene expression [12,24]. Contrary to plant ETI signaling, the outcomes of plant PTI signaling were enhanced during brief exposure to moderately elevated temperatures [25,26]. Cheng and colleagues revealed that in response to flg22 (a 22-amino acid peptide of bacterial flagellin), expression of the PTI marker genes *WRKY29* and *FRK1*, MAPKs activation, and BIK1 phosphorylation at a relatively high ambient temperature occurred faster and stronger than that at a low temperature [25]. The different outcomes of plant immune signaling response to temperature oscillations suggest that plant immune layers may respond to temperatures in different manners [27].

Previous efforts revealed that temperature fluctuation has an obvious effect on the disease incidence caused by the ascomycetous fungus *L. theobromae* [28]. Although there are a couple of examples of studies where the transcriptome of *L. theobromae* under different temperatures was compared [29], research efforts focused on co-measuring gene transcriptional variations of the plant and pathogen in response to temperature changes were barely reported, and therefore, how variation in plant gene transcription is related to the variation in its pathogen partner gene transcription is not understood at the mechanistic level. Here, we establish a rigorous experiment design that comprises one grapevine cultivar inoculated with the *L. theobromae* strain under three temperatures and two experimental controls (mock-inoculated grapevine and cultured fungus). We perform a dual RNA-seq with systemic transcriptome comparisons to detect grapevine and *L. theobromae* gene modules that are co-transcribed at different temperatures and identify a set of key genes that are transcriptionally responsive to temperature fluctuations. Our findings reveal that temperature fluctuation has a crucial role in shaping the outcome of plant–pathogen interaction and drive further explorations of the molecular mechanisms that underlie temperature-mediated pathogen invasion and host evasion. 

## 2. Materials and Methods

### 2.1. Fungus, Plant Materials and Culture Conditions

The strain *L. theobromae* was cultured on complete media (CM, 6 g yeast extract, 3 g casein acid hydrolysate, 3 g casein enzymatic hydrolysate, and 10 g sucrose per liter, all reagents were purchased from Sigma-Aldrich, St. Louis, MO, USA) and maintained at 28 °C under equal light and dark cycles. The green shoots of *Vitis vinifera* cv ‘Summer Black’ used for inoculation were collected from a Xiangyi field vineyard in Shunyi, Beijing, China. The obtained grapevine shoots were inoculated with mycelial plugs (5 mm in diameter), followed by incubation in a chamber with constant temperature and humidity. Diseased tissues used for RNA extraction were collected at 24, 48, and 72 h post-inoculation (hpi).

### 2.2. Pathogenicity Tests on Detached Green Shoots

For pathogenicity tests, *L. theobromae* strain CSS-01s mycelial plugs (5 mm in diameter) were collected and inoculated on wounded grapevine shoots of 1-year-old susceptible *Vitis vinifera* cv. ‘Summer Black’, followed by incubation in a chamber with constant humidity and different temperatures (25 °C and 35 °C). Diseased grapevine shoots were photographed 3 days post-inoculation (dpi). Significant differences were evaluated using the one-way analysis of variance (ANOVA) and least significant difference (LSD) tests (** α = 0.01; * α = 0.05).

### 2.3. Library Construction and Dual RNA-Seq

Total RNA was isolated using the TRIzol agent (Invitrogen, Carlsbad, CA, USA) according to the manufacturer’s instructions. The integrity, purity, and concentration of isolated RNA were evaluated using an Agilent 2100 Bioanalyzer (Agilent Technologies, Palo Alto, CA, USA) and a NanoDrop 2000 spectrophotometer (Thermo Fisher Scientific, Waltham, MA, USA). The messenger RNA (mRNA) purification, end repair, adapter ligation, and cDNA amplification were performed to prepare Illumina RNA-seq libraries. The final quantified libraries were sequenced on an Illumina NovaSeq 6000 platform (Illumina, San Diego, CA, USA) of Novogene Co., Ltd. (Beijing, China). Quality control of raw data was processed by running the Fastp program (version 0.19.7), and low-quality sequences (phred ≤ 20 accounting for over 50%, adapter and poly-N contamination) were filtered. The high-quality reads were aligned to the reference genomes of grapevine (*V. vinifera* var. “PN40024”) and *L. theobromae* using the HISAT2 software (version 2.1.0). 

### 2.4. Gene Co-Transcribed Network between Grapevine and L. theobromae

The weighted gene co-expression network analysis (WGCNA) was adopted to separately build the grapevine and *L. theobromae* modules in which one gene cluster displayed a similar correlated transcription pattern [30]. As we were interested in *L. theobromae* genes responsive to fluctuating temperatures, the pool of *L. theobromae* genes that were significantly expressed under different temperatures was used to build fungal gene modules, and vice versa for grapevine genes. 

The association of modules between two organisms was calculated by measuring the Pearson correlation between grapevine module eigengenes and *L. theobromae* module eigengenes using the default WGCNA ‘relating modules to external information’ analysis [30]. Moreover, we calculated the gene significance (Pearson correlation) for each gene to the corresponding correlated modules and the connectivity (membership) of each gene to its module according to the methods described by Mateus and colleagues [30]. Based on this information, we obtained a set of ‘key genes’ that were highly correlated to the modules of the counterpart partner organism (in the top 10% quantile) and exhibited a high module membership value (>0.8). 

### 2.5. Gene Ontology (GO) Enrichment Analyses

Gene ontology analyses, functional classification, and inferred pathway diagram Gene Ontology (GO) enrichment analyses for the key genes within grapevine and *L. theobromae* modules were performed with their reference genome resources. The GO enrichment analyses of differentially expressed genes (DEGs) were performed by the clusterProfiler R package. GO terms with adjusted *p*-values < 0.05 were considered significantly enriched DEGs.

### 2.6. Real-Time qPCR Analyses

To substantiate the reliability of RNA-seq data, a total of 12 randomly selected key genes belonging to different gene modules were selected for transcription assessment via reverse-transcriptase quantitative PCR (RT-qPCR) assays. Briefly, total RNA was extracted from the experimental samples using the TRIzol agent according to the manufacturer’s instruction (Invitrogen, Carlsbad, CA, USA), and then was reverse-transcribed into cDNA using the TransScript^®^ One-Step gDNA Removal and cDNA Synthesis SuperMix Kit (TransGen Biotech, Beijing, China). The qRT-PCR tests were performed using an ABI 7500 Real-Time system (Applied Biosystems, Waltham, MA, USA) and conducted in a 20 μL volume mixture comprising 10 μL RealStar Green Fast Mixture with ROX II (GenStar Biosolutions, Beijing, China), 1.0 μL cDNA, 0.2 μM primer, and 8.2 μL sterile ddH_2_O. The PCR programs were progressed as follows: denaturation at 95 °C for 2 min, followed by 40 cycles of 95 °C for 15 s and 60 °C for 30 s. The actin genes were used as the controls. The relative abundances of tested transcripts were calculated using the 2^−ΔΔCT^ method to assess their relative expression levels [31]. All experiments were repeated thrice independently, and the primers used for PCR assays were as listed in Table S1.

## 3. Results

### 3.1. Disease Development under Different Temperatures

**Figure 1:**
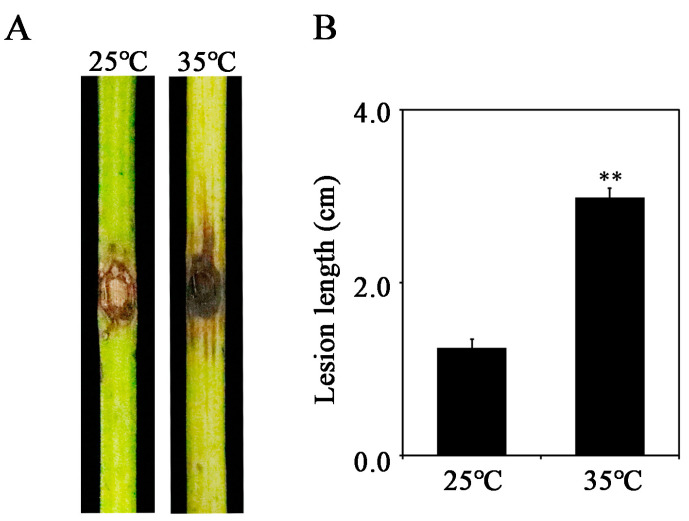
The influence of temperature on the virulence of *Lasiodiplodia theobromae*. (**A**) Pathogenicity tests of *L. theobromae* under different temperatures. One-year-old grapevine shoots collected from the vineyard were inoculated with mycelial plugs (5 mm in diameter), followed by incubation inside a chamber with different temperatures (25 °C and 35 °C) and constant humidity. The inoculated grapevines were photographed at 3 days post inoculation (dpi). (**B**) Statistical analyses of lesion length caused by *L. theobromae* under different temperatures. The means and standard errors were calculated from five replicates, and significant differences are indicated by stars. Significant differences were evaluated using the one-way analysis of variance (ANOVA) and least significant difference (LSD) tests (** α = 0.01).

To evaluate whether temperature affects the occurrence and development of grapevine canker disease caused by *L. theobromae*, we inoculated the mycelial plugs of the fungus on detached grapevine green shoots under two temperatures (25 °C and 35 °C) and measured the diseased areas at 3 dpi. The pathogenicity tests reveal that *L. theobromae* infection under a relatively higher temperature (35 °C) results in larger diseased areas compared to that under a lower temperature (25 °C) (Figure 1), indicating that high temperature pronouncedly promotes disease development in grapevine caused by *L. theobromae*. 

To further investigate the underlying mechanism by which grapevine and *L. theobromae* respond to temperature oscillation, we profiled their transcriptomes using a dual RNA-seq approach with RNA isolated from grapevines infected by *L. theobromae* under different temperatures (25 °C, 30 °C, and 35 °C). Grapevine without inoculation and *L. theobromae* cultured on CM media were used as the controls, and thereby, a total of 81 group samples were prepared for RNA-seq. The filtrated reads were mapped to the reference genomes of grapevine (*Vitis vinifera* var. “PN40024”) and *L. theobromae* (NCBI SAMN08892999). The sequence data reported in the current paper have been deposited in the Genome Sequence Archive [32] of the National Genomics Data Center [33], China National Center for Bioinformation/Beijing Institute of Genomics, Chinese Academy of Sciences (GSA accession number CRA013199 and CRA013200) and are publicly accessible at https://ngdc.cncb.ac.cn/gsa, accessed on 5 December 2023. In terms of the grapevine infected by *L. theobromae*, an average of 54.80 million reads were generated, and approximately 80% of filtrated reads were mapped to the grapevine genome. Comparatively, the percentage of filtrated reads that were mapped to the *L. theobromae* genome, however, was less than 2% (Table S2).

### 3.2. Identification of Grapevine and Fungal Gene Modules That Are Correlated with Infection Time and Temperature

**Figure 2:**
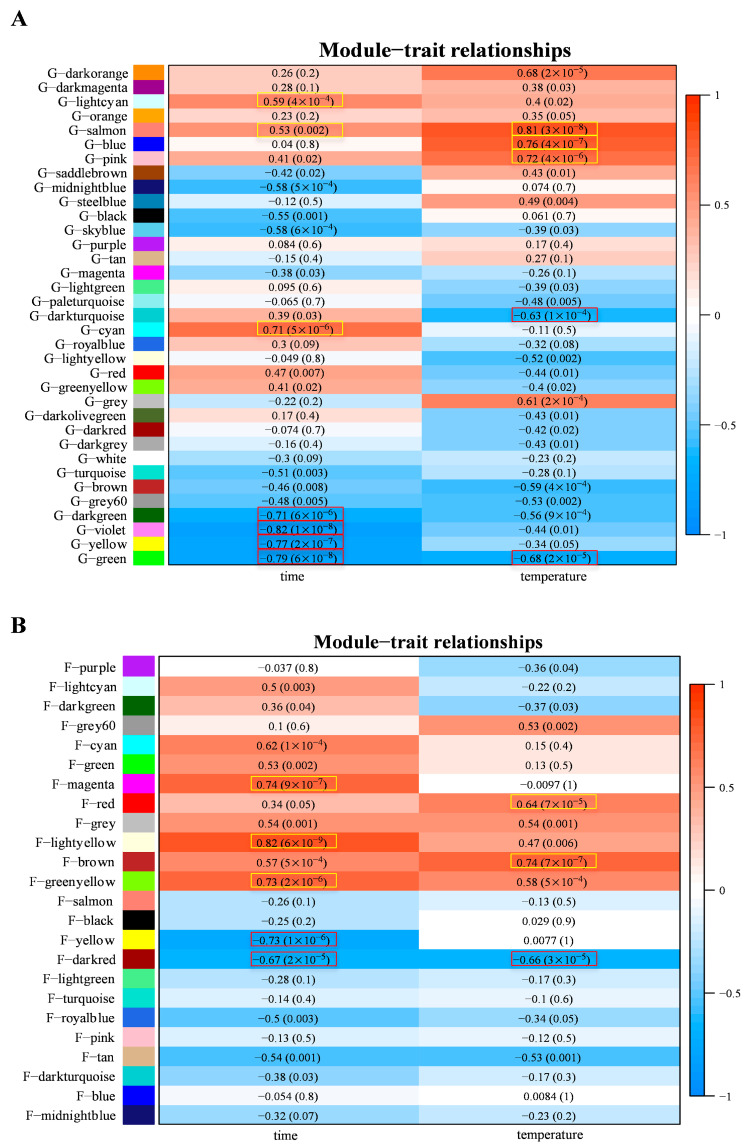
Correlation of grapevine and *L. theobromae* gene modules with infection time and temperature. (**A**) Correlation matrix between grapevine modules (rows) with infection time and temperature (columns). (**B**) Correlation matrix between *L. theobromae* gene modules (rows) with infection time and temperature (columns). The Pearson coefficient and *p*-value were reported. Highly positive correlations and highly negative correlations were marked with yellow boxes and red boxes, respectively.

The measurement of plant and fungus gene transcript abundance, together with its transcription variance caused by different treatments within the same experiment, allows us to identify the key genes that are highly correlated with environmental factors during the infection. The gene transcription differences that arose from different treatments were analyzed to identify the co-transcribed grapevine and *L. theobromae* gene clusters. Based on the weighted gene co-expression network analysis (WGCNA), we identified 35 and 24 co-transcribed gene modules from grapevine and *L. theobromae*, respectively, and the correlation matrix between all of the grapevine modules and all of the *L. theobromae* modules was constructed (Figure S1). Additionally, the correlation of identified grapevine modules and fungal modules to temperature and infection time was also evaluated and presented in Figure 2. Within the correlation matrix, highly positive correlations and highly negative correlations were marked with yellow boxes and red boxes, respectively (Figure 2). 

### 3.3. Gene Co-Transcribed Networks as an Efficient Tool to Decipher the Molecular Interactions between Grapevine and L. theobromae

**Figure 3:**
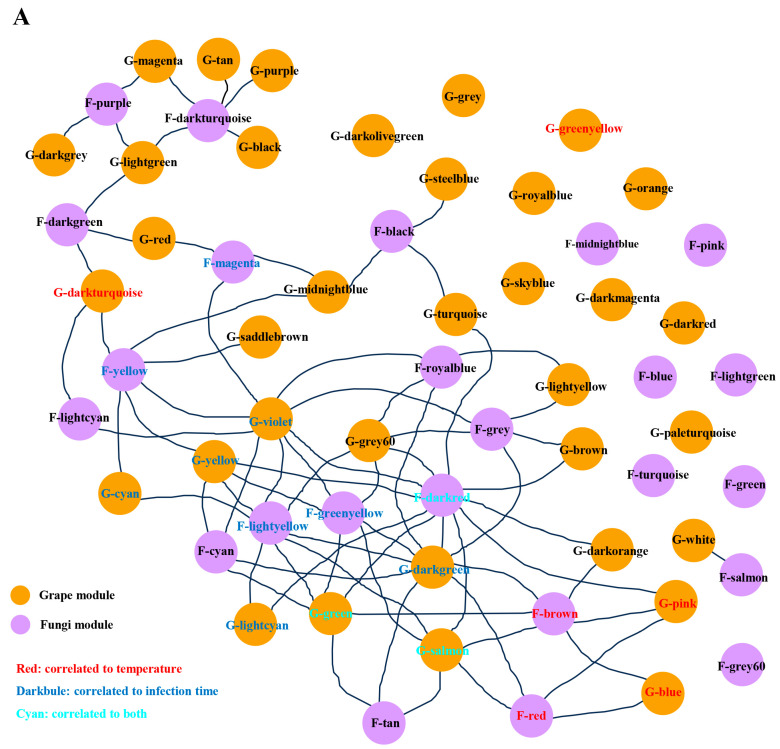

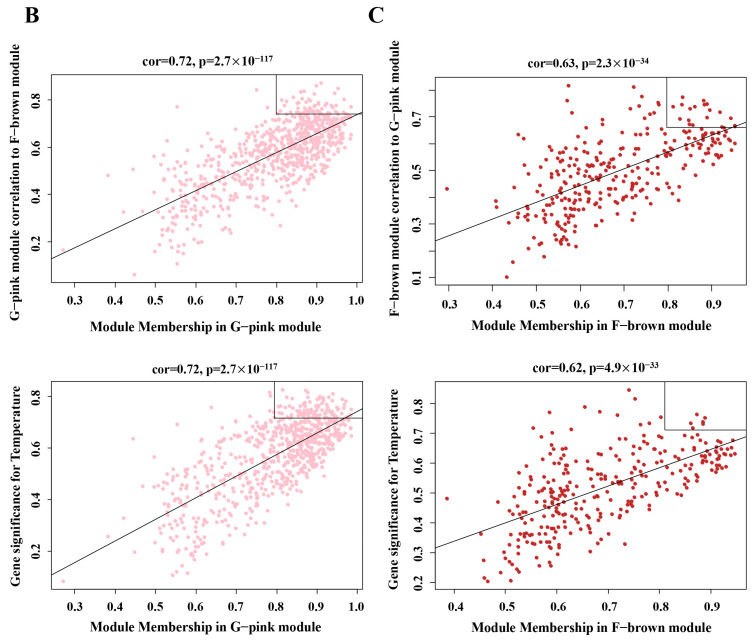
Gene co-expression network analyses. (**A**) Web of grapevine and *L. theobromae* modules. Highly correlated modules were linked with a line. The grapevine modules and fungus modules are marked in yellow and purple colors, respectively. (**B**,**C**) Examples of key gene identification between grapevine and *L. theobromae* gene modules. Each dot represents one gene, and the key genes are marked with a black box. The key genes were defined as those that most correlated to the modules of the partner organism (top 10% quantile) and exhibited a high module membership (>0.8).

In the current study, we evaluated the co-orchestrated interaction between grapevine and *L. theobromae* by identifying genes of grapevine and *L. theobromae* that were highly correlated based on the released transcription data. Among these identified modules mentioned above, a total of 26 grapevine modules and 17 fungal modules were correlated to over one module of the partner species (Figure 3A). Within these inter-correlated modules, we found that five modules (three grapevine modules and two fungus modules) were merely correlated to temperature but not to infection time. Additionally, a total of nine modules (five grapevine modules and four fungus modules) were only correlated to infection time but not to temperature. Moreover, only three modules (two grapevine modules and one fungus module) were correlated to both temperature and infection time (Figure 3A). Currently, we mainly focus on identifying temperature-responsive genes involved in the interplay of grapevine and *L. theobromae*, and therefore, temperature-responsive genes within grapevine and fungal modules were further excavated.

### 3.4. Grapevine and L. theobromae Gene Modules Correlated to Temperature

**Figure 4:**
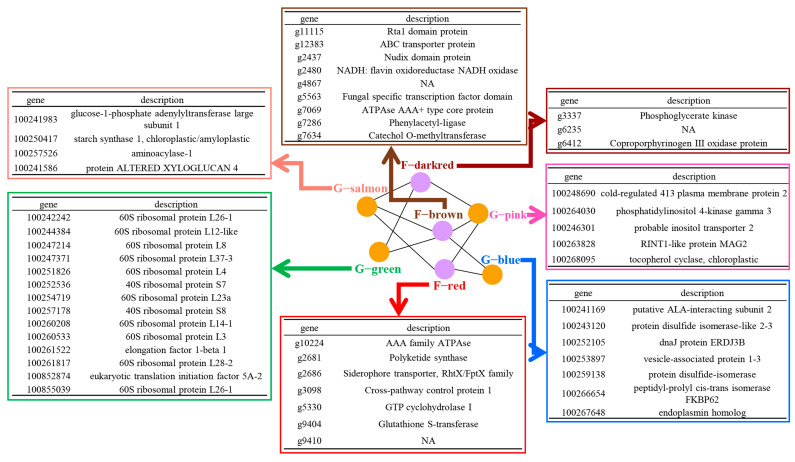
Function annotation of enriched temperature-responsive genes within grapevine and *L. theobromae* modules. G-salmon, G-green, G-pink, and G-blue indicate four grapevine gene modules. F-dark red, F-brown, and F-red mark three *L. theobromae* gene modules.

To detect the key genes that were correlated to temperature, we originally evaluated the gene’s module membership within the module that was correlated to a partner module and the gene’s correlation to the partner module and temperature. Examples of correlation between one module with its partner module are shown in Figure 3B,C. This evaluation allowed us to identify some key genes within the given modules (Figures S2–S21). Within all of the grapevine and *L. theobromae* correlated gene modules, we identified 175 grapevine key genes and 19 fungal key genes that were highly representative of their modules and correlated to their partner gene modules as well as temperature (Tables S3 and S4). Based on the Gene Ontology (GO) enrichment analyses, it was found that most enriched terms of temperature-correlated genes are involved in carbohydrate and peptide metabolism, stress response, and protein folding in grapevine. In *L. theobromae*, the enriched genes mainly participated in the metabolic process of porphyrin-containing compounds, pteridine-containing compounds, and glucose (Figure 4). These results suggest that multiple biological processes inside grapevine and *L. theobromae* cells were associated with environmental temperature changes.

### 3.5. Transcription Analyses of Temperature-Responsive Genes within Grapevine and L. theobromae Gene Modules

**Figure 5:**
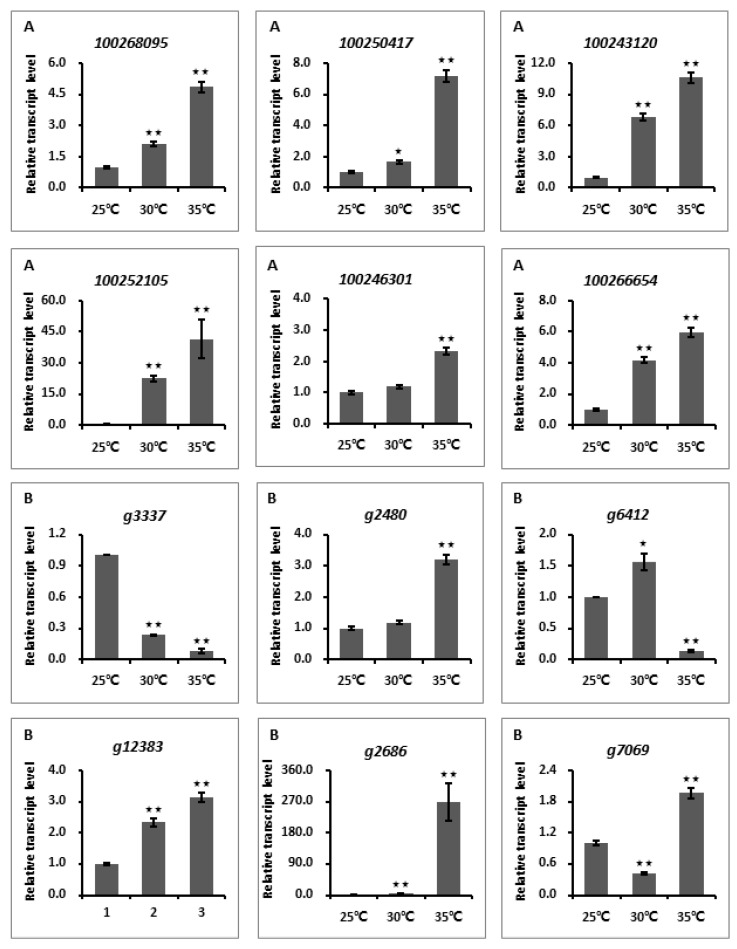
Transcription analyses of temperature-responsive genes within grapevine and *L. theobromae* modules by reverse-transcriptase quantitative PCR. Boxes labeled with (**A**,**B**) indicate the genes were randomly selected from grapevine and *L. theobromae*, respectively. Relative transcript abundances of tested genes were calculated using the 2^−ΔΔCT^ method. The relative transcript levels of each gene under different temperatures were normalized by the actin gene and calibrated against that of 25 °C. The assays were performed with three independent biological repetitions and three replicates each. A representative set of data is presented. The data are means ± standard error, and significant differences are marked with asterisks (^★★^ α = 0.01; ^★^ α = 0.05).

To confirm the reliability of our RNA-seq data, the transcript abundances of 12 randomly selected genes (six genes from grapevine modules and six genes from *L. theobromae* modules) were tested by RT-qPCR. The experimental results revealed that the relative transcript levels of two genes (*g3337* and *g6412*) were significantly decreased under high temperature (35 °C) in comparison with that of low temperature (25 °C), and that the other 10 genes, however, were transcriptionally up-regulated under higher temperatures (Figure 5), suggesting that these genes are indeed temperature-responsive factors.

## 4. Discussion

In terms of *L. theobromae*, a set of omics researches have been performed to decipher the underlying mechanism by which *L. theobromae* infects and colonizes plant tissues [7,29,34,35,36]. Previous efforts, however, mainly focused on profiling pathogen or plant transcriptome variation before and after *L. theobromae* infection. To some extent, it is limited and biased to dissect the molecular mechanism of disease development if just a single factor within the ‘disease triangle’ is taken into account during plant–pathogen interaction that captures only a fraction of the dynamic plant–pathogen–temperature interactions that occur in nature. In this study, we explored gene transcription patterns of grapevine and *L. theobromae* in response to temperature oscillations. The main novelty of the research is that we systemically and simultaneously analyzed the co-transcription network of plant and pathogen in the context of considering environmental factors within the ‘disease triangle’, which allowed us to detect a substantial body of quantitative differences in gene transcription levels and screen a set of key genes that are highly correlated between the two organisms, as well as correlated to temperature changes. Such detection of the plant–*L. theobromae* gene network and key genes that are involved in temperature-mediated disease development has not been documented in previous research.

Concerning the RNA-seq data, the ratio of obtained reads that were mapped to the grapevine genome reached up to 80%. However, the ratio of obtained reads that were mapped to the genome of *L. theobromae* was extremely low, less than 2%, which was similar to the mapped data reported by another research group [35]. The low percentage of mapped reads of the *L. theobromae* RNA-seq dataset may be associated with the fact that within the infected grapevine tissues, the amount of invasive mycelium is extremely low relative to the amount of grapevine tissue infected by *L. theobromae*. A couple of researchers have adopted some other experiment protocols in which fungus was cultured within liquid media added with exogenous ingredients such as carboxymethylcellulose or chips of grapevine wood to mimic actual plant–pathogen interplay states [37,38,39]. These methods are capable of overcoming the intractable issues we encountered in this study, but they will cause other trouble, as the fungal mycelia cultured within artificial media do not resemble the actual infection states.

It is well-known that a plethora of, if not all, plant–pathogen interactions are profoundly influenced by external environmental conditions including temperature oscillations [12]. The long-standing ‘disease triangle’ dogma in plant pathology states the requirement of optimal environmental conditions for the development of plant disease, in addition to a genetically susceptible host and a genetically virulent pathogen [12,26,40]. In many plant–pathogen interaction systems, such as rice–*Magnaporthe oryzae* [41], Arabidopsis–*Pst* DC3000 [21], tobacco–Tobacco mosaic virus (TMV) [14], and tomato–root-knot nematode [42], the molecular mechanisms underlying temperature-mediated plant–pathogen interplay have been extensively explored. However, the molecular mechanism of how grapevine responds to *L. theobromae* remains to be elucidated. In this study, we first tested and verified that high temperature is conducive to the disease development caused by *L. theobromae*, which is in accord with the results of previous reports [28,43]. 

Dual RNA-seq has been accepted as an efficient tool that allows researchers to investigate the interaction between plants and microbes [30,35]. Based on RNA-seq data, we preliminarily clustered the gene transcript of grapevine and *L. theobromae* and identified correlated expression patterns between both organisms, as well as with temperature oscillation and infection time. Here, we mainly focused on dissecting the correlated gene modules of grapevine and *L. theobromae* that are responsive to temperature fluctuations. According to the bioinformatic analyses, an extensively correlated web of co-expressed gene modules between the two organisms was revealed. Within the correlated gene networks, we finally identified a set of key genes that were assumed to be involved in the temperature-mediated plant–pathogen interaction. In grapevine, these key genes are predicted to play important roles in multiple cellular processes, including protein folding, stress response regulation, and carbohydrate and peptide metabolism, which suggests these biological processes may be interconnected with temperature-mediated plant immunity. In the pathogen, identified genes were predicted to be involved in the metabolic processes of porphyrin- and pteridine-containing compounds and glucose. The production of porphyrin in *Propionibacterium acnes* was temperature-dependent [44], suggesting that transcription reprogramming of related genes involved in porphyrin-containing compound metabolism may be an adaptive strategy adopted by *L. theobromae* within the context of frequent and extreme heat weave events during the co-evolution process, although this assumption remains to be substantiated. This study reveals intense correlations among the identified genes that are assumed to participate in the metabolism of many compounds and stress response regulation, and these biological processes are probably co-orchestrated during temperature modulation of plant–microbe interaction.

Altogether, although we are fully aware of the substantial advances that have been made in explaining the pathogenic mechanism of *L. theobromae* [1,29,36,45], our study systemically deciphered the transcriptional networks that co-correlated both plant and pathogen organisms simultaneously and screened dozens of key genes that respond to temperature fluctuation. Further exploration of the molecular function of these genes will be conducive to comprehensively shedding light on the underlying mechanisms of how grapevine and *L. theobromae* rigorously integrated the interconnected signals when high- or low-temperature events occurred in nature.

## 5. Conclusions and Outlook

In this study, we identified a total of 26 grapevine gene modules and 17 fungal gene modules that were correlated to at least one gene module of the partner species and screened a set of temperature-responsive genes within these given modules. These findings supply valuable genetic resources for further elucidation of the underlying mechanism of temperature-mediated disease occurrence and open a possibility for the development of temperature-resilient and disease-resistant grapevines.

## Data Availability

The data that support the findings of this study are available in the Supplementary Materials of this article.

## Supplementary Materials

The following supporting information can be downloaded at: https://www.mdpi.com/article/10.3390/jof9121197/s1, Figure S1. Overview of grapevine and *L. theobromae* module correlations to each other. The correlation coefficient and its *p*-value were displayed for each module–module correlation. Highly positive correlations and negative correlations were marked with yellow and red boxes, respectively. Figure S2. Gene Ontology analyses of key genes within grapevine G-pink module that are correlated with *L. theobromae* F-brown module and temperature. Figure S3. Gene Ontology analyses of key genes within *L. theobromae* F-brown module that are correlated with grapevine G-pink module and temperature. Figure S4. Gene Ontology analyses of key genes within grapevine G-pink module that are correlated with *L. theobromae* F-red module and temperature. Figure S5. Gene Ontology analyses of key genes within *L. theobromae* F-red module that are correlated with grapevine G-pink module and temperature. Figure S6. Gene Ontology analyses of key genes within grapevine G-pink module that are correlated with *L. theobromae* F-dark red module and temperature. Figure S7. Gene Ontology analyses of key genes within *L. theobromae* F-dark red module that are correlated with grapevine G-pink module and temperature. Figure S8. Gene Ontology analyses of key genes within grapevine G-blue module that are correlated with *L. theobromae* F-red module and temperature. Figure S9. Gene Ontology analyses of key genes within *L. theobromae* F-red module that are correlated with grapevine G-blue module and temperature. Figure S10. Gene Ontology analyses of key genes within grapevine G-blue module that are correlated with *L. theobromae* F-brown module and temperature. Figure S11. Gene Ontology analyses of key genes within *L. theobromae* F-brown module that are correlated with grapevine G-blue module and temperature. Figure S12. Gene Ontology analyses of key genes within grapevine G-salmon module that are correlated with *L. theobromae* F-red module and temperature. Figure S13. Gene Ontology analyses of key genes within *L. theobromae* F-red module that are correlated with grapevine G-salmon module and temperature. Figure S14. Gene Ontology analyses of key genes within grapevine G-salmon module that are correlated with *L. theobromae* F-brown and temperature. Figure S15. Gene Ontology analyses of key genes within *L. theobromae* F-brown module that are correlated with grapevine G-salmon module and temperature. Figure S16. Gene Ontology analyses of key genes within grapevine G-salmon module that are correlated with *L. theobromae* F-dark red module and temperature. Figure S17. Gene Ontology analyses of key genes within *L. theobromae* F-dark red module that are correlated with grapevine G-salmon module and temperature. Figure S18. Gene Ontology analyses of key genes within grapevine G-green module that are correlated with *L. theobromae* F-brown module and temperature. Figure S19. Gene Ontology analyses of key genes within *L. theobromae* F-brown module that are correlated with grapevine G-green module and temperature. Figure S20. Gene Ontology analyses of key genes within grapevine G-green module that are correlated with *L. theobromae* F-dark red module and temperature. Figure S21. Gene Ontology analyses of key genes within *L. theobromae* F-dark red module that are correlated with grapevine G-green module and temperature. Table S1. Primers used in the study. Table S2. Summary results of the sequencing reads of grapevine samples infected by *L. theobromae*. Table S3. List of grapevine temperature-correlated genes found in correlated modules between grapevine and *L. theobromae*. Table S4. List of *L. theobromae* temperature-correlated genes found in correlated modules between grapevine and *L. theobromae*.
