# Supplementary material for: Dual RNA-Seq Reveals Temperature-Mediated Gene Reprogramming and Molecular Crosstalk between Grapevine and Lasiodiplodia theobromae
Source: J Fungi (Basel). 2023 Dec 14;9(12):1197. doi: 10.3390/jof9121197 (PMC10745131; doi:10.3390/jof9121197)
Supplement: Supplementary file 1 [file jof-09-01197-s001.zip › Supplementary Figure.pdf]

Fig. S1 Overview of grapevine and *L. theobromae* modules correlation to each other.

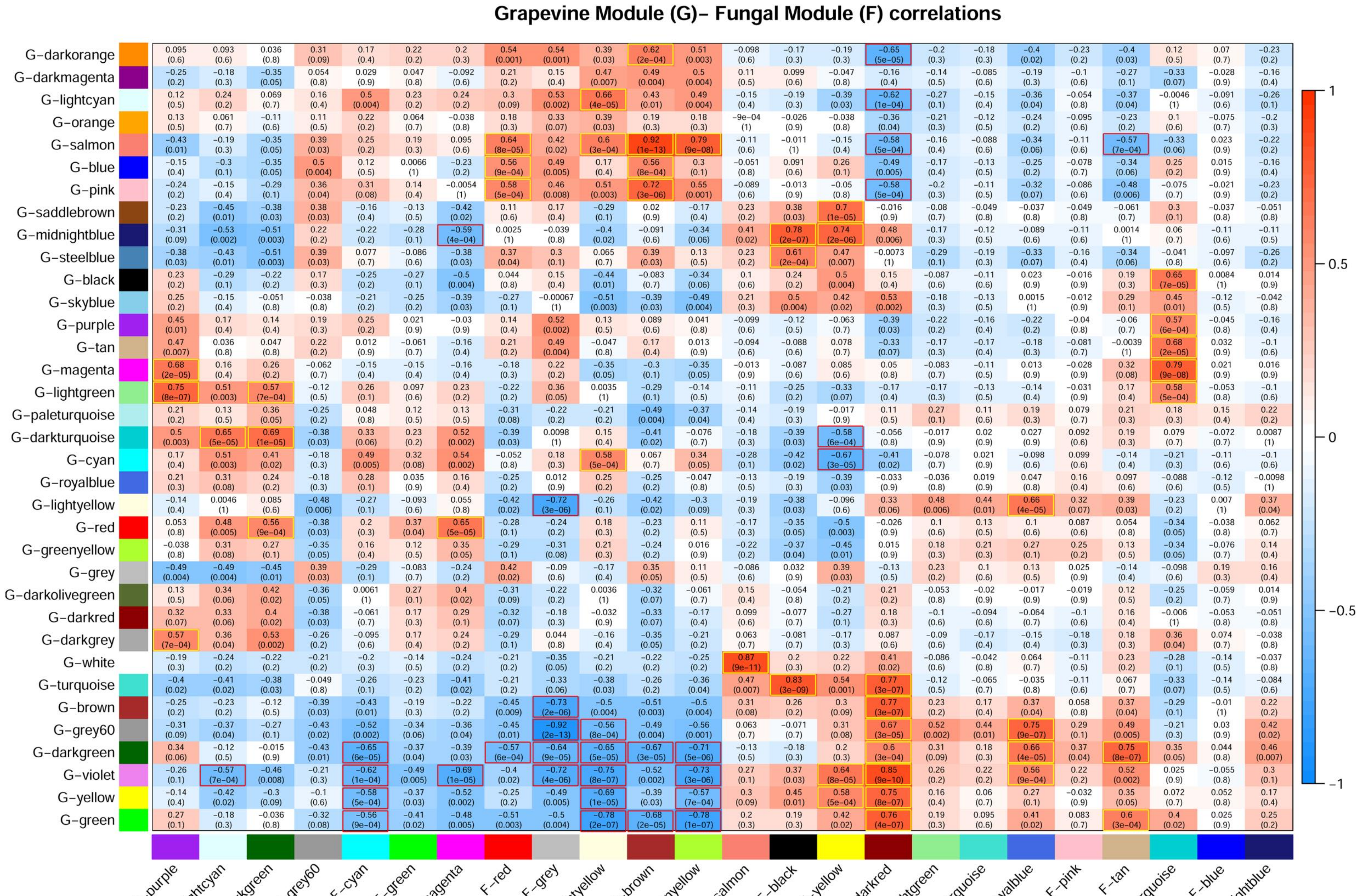

Fig. S2 Gene Ontology analyses of key genes within grapevine G−pink module that are correlated with *L. theobromae* F−brown module and temperature.

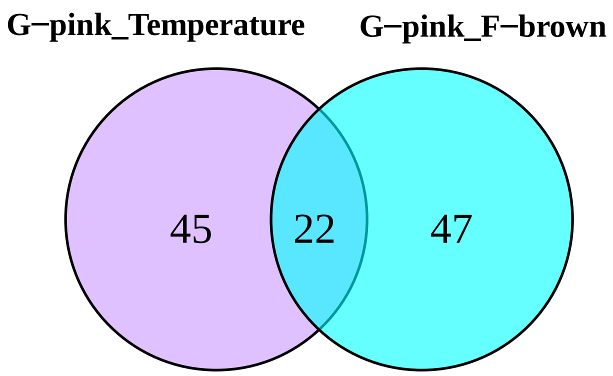

Common_hub_gene_G−pink_F−brown_Temp_GO_enrich

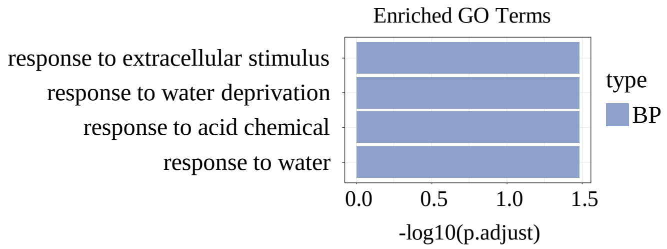

Common_hub_gene_G−pink_F−brown_Temp_anno

	gene	description
1	100232889	ripening-related bZIP protein-like
2	100233076	glucan endo-1, 3-beta-glucosidase
3	100244508	adagio protein 1
4	100245170	O-fucosyltransferase 15
5	100246781	cytochrome P450 71A3
6	100247186	protein detoxification 27
7	100247368	uncharacterized LOC100247368
8	100248690	cold-regulated 413 plasma membrane protein 2
9	100251651	uncharacterized LOC100251651
10	100253180	CBL-interacting protein kinase 04
11	100260954	protein MEI2-like 4
12	100261257	cysteine proteinase COT44
13	100262654	sec1 family domain-containing protein MIP3
14	100264030	phosphatidylinositol 4-kinase gamma 3
15	100264466	uncharacterized LOC100264466
16	100265388	putative BPI/LBP family protein At1g04970
17	100265471	probable xyloglucan endotransglucosylase/hydrolase protein 32
18	100266331	aldehyde dehydrogenase family 3 member H1
19	100266362	Paramyosin
20	100266572	glycine-rich domain-containing protein 2
21	100268095	tocopherol cyclase, chloroplastic
22	novel.408	-

Fig. S3 Gene Ontology analyses of key genes within *L. theobromae* F−brown module that are correlated with grapevine G−pink module and temperature.

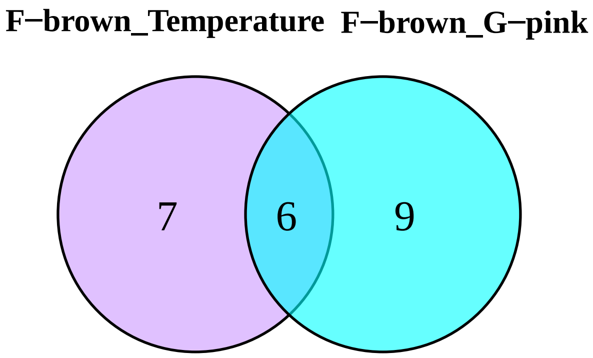

Common_hub_gene_F−brown_G−pink_Temp_GO_enrich

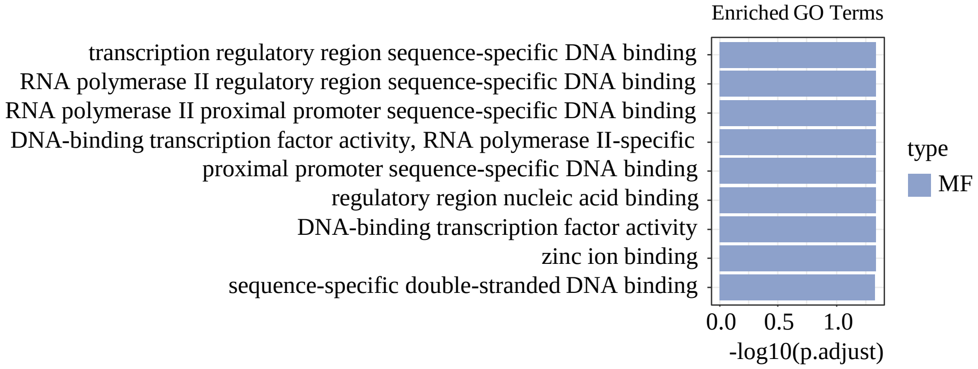

Common_hub_gene_F−brown_G−pink_Temp_anno

	gene	description
1	g5563	Fungal specific transcription factor domain
2	g7634	O-methyltransferase
3	g12383	ABC transporter protein
4	g2437	Nudix domain protein
5	g2480	NADH: flavin oxidoreductase NADH oxidase
6	g7286	Phenylacetyl-ligase

Fig. S4 Gene Ontology analyses of key genes within grapevine G−pink module that are correlated with *L. theobromae* F−red module and temperature.

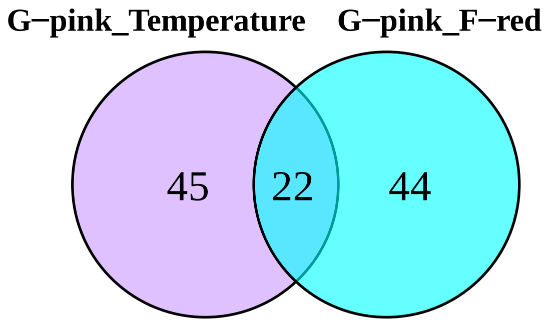

Common_hub_gene_G−pink_F−red_Temp_anno

	gene	description
1	100232889	ripening-related bZIP protein-like
2	100244508	adagio protein 1
3	100246781	cytochrome P450 71A3
4	100247186	protein detoxification 27
5	100247368	uncharacterized LOC100247368
6	100248696	autophagy-related protein 8i
7	100253180	CBL-interacting protein kinase 04
8	100256125	uncharacterized LOC100256125
9	100260954	protein MEI2-like 4
10	100261030	protein MEI2-like 4
11	100262863	profilin-1
12	100262931	uncharacterized LOC100262931
13	100264190	WD repeat-containing protein 44
14	100264466	uncharacterized LOC100264466
15	100265471	probable xyloglucan endotransglucosylase/hydrolase protein 32
16	100265815	vacuolar amino acid transporter 1
17	100266050	GABA transporter 1
18	100266130	box C/D snoRNA protein 1
19	100266362	Paramyosin
20	100266572	glycine-rich domain-containing protein 2
21	104877310	beta-glucosidase 18
22	novel.408	-

Fig. S5 Gene Ontology analyses of key genes within *L. theobromae* F−red module that are correlated with grapevine G−pink module and temperature.

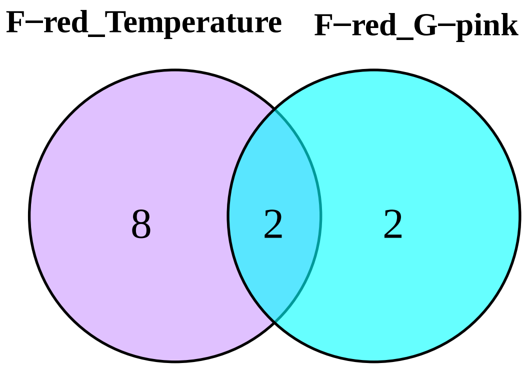

Common_hub_gene_F−red_G−pink_Temp_anno

	gene	description
1	g5330	GTP cyclohydrolase I
2	g9404	Glutathione S-transferase

Fig. S6 Gene Ontology analyses of key genes within grapevine G−pink module that are correlated with *L. theobromae* F−darkred module and temperature.

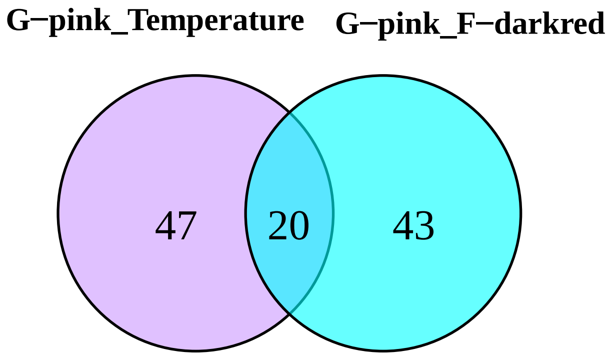

Common_hub_gene_G−pink_F−darkred_Temp_GO_enrich

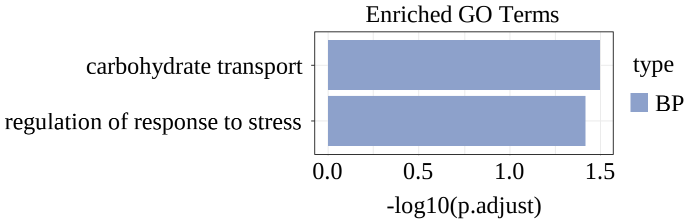

Common_hub_gene_G−pink_F−darkred_Temp_anno

	gene	description
1	100244508	adagio protein 1
2	100246301	probable inositol transporter 2
3	100247561	deoxyhypusine synthase
4	100248377	omega-6 fatty acid desaturase, chloroplastic
5	100249560	AT-hook motif nuclear-localized protein 5
6	100250069	protein ENHANCED DISEASE RESISTANCE 2-like
7	100251651	uncharacterized LOC100251651
8	100254964	cysteine-rich and transmembrane domain-containing protein A
9	100255560	LRR receptor-like serine/threonine-protein kinase GSO2
10	100256125	uncharacterized LOC100256125
11	100259117	uncharacterized LOC100259117
12	100260954	protein MEI2-like 4
13	100261030	protein MEI2-like 4
14	100261257	cysteine proteinase COT44
15	100263828	RINT1-like protein MAG2
16	100264466	uncharacterized LOC100264466
17	100265388	putative BPI/LBP family protein At1g04970
18	100265815	vacuolar amino acid transporter 1
19	100266362	paramyosin
20	100268095	tocopherol cyclase, chloroplastic

Fig. S7 Gene Ontology analyses of key genes within *L. theobromae* F−darkred module that are correlated with grapevine G−pink module and temperature.

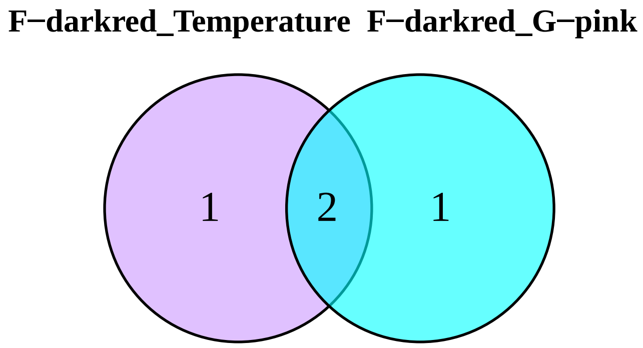

Common_hub_gene_F−darkred_G−pink_Temp_GO_enrich

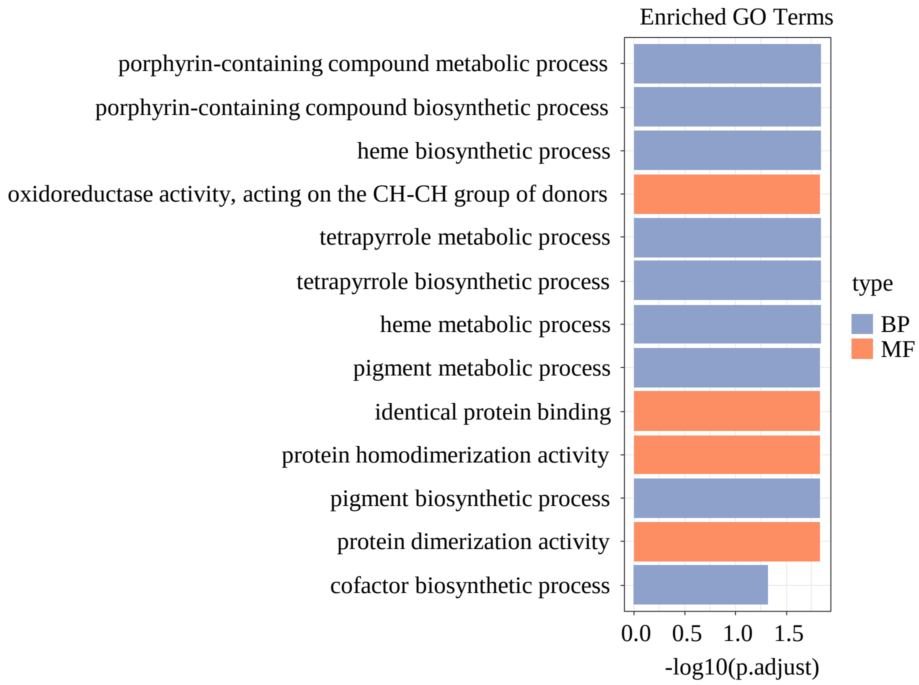

Common_hub_gene_F−darkred_G−pink_Temp_anno

	gene	Description
1	g6235	NA
2	g6412	Coproporphyrinogen III oxidase protein

Fig. S8 Gene Ontology analyses of key genes within grapevine G−blue module that are correlated with *L. theobromae* F−red module and temperature.

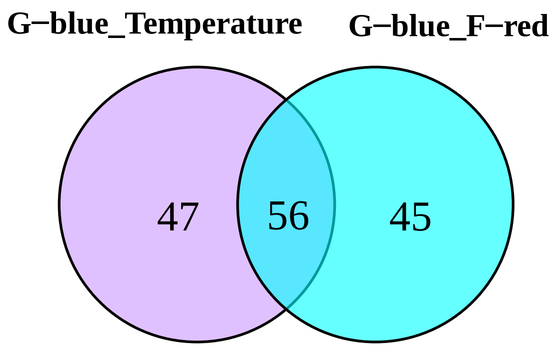

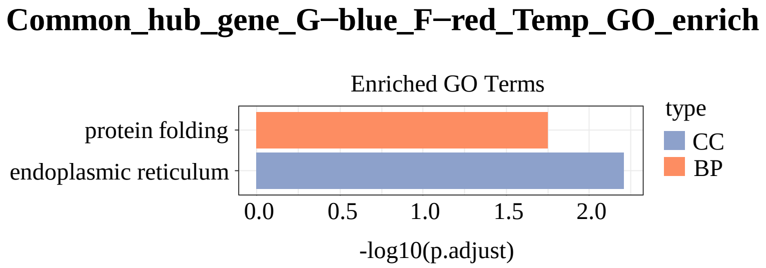

Common_hub_gene_G−blue_F−red_Temp_anno

	gene	description
1	100241091	nudix hydrolase 14, chloroplastic-like
2	100241158	60S ribosomal protein L32-1
3	100241169	putative ALA-interacting subunit 2
4	100241270	putative pentatricopeptide repeat-containing protein At1g09680
5	100242205	protein POLLEN DEFECTIVE IN GUIDANCE 1
6	100243115	protein translation factor SUI1 homolog 1
7	100243120	protein disulfide isomerase-like 2-3
8	100243248	transcription factor RF2b
9	100244643	14-3-3-like protein B
10	100246124	probable pectate lyase 12-like
11	100246648	RNA-binding protein 42
12	100247016	uncharacterized protein At1g04910
13	100247365	GTP-binding nuclear protein Ran-3
14	100248135	calcyclin-binding protein
15	100248504	PRA1 family protein E
16	100248687	2-hydroxyisoflavanone dehydratase
17	100248914	chaperone protein DnaJ
18	100249097	peptidyl-prolyl cis-trans isomerase FKBP62
19	100249249	multiprotein-bridging factor 1c
20	100249262	uncharacterized LOC100249262
21	100249761	uncharacterized protein YwbO
22	100250042	non-specific lipid-transfer protein-like
23	100250368	FAD synthase
24	100250629	selenoprotein K-like
25	100251534	probable inactive ATP-dependent zinc metalloprotease FTSHI 2, chloroplastic
26	100252105	dnaJ protein ERDJ3B
27	100252468	bifunctional purple acid phosphatase 26
28	100252886	purple acid phosphatase 23
29	100253897	vesicle-associated protein 1-3
30	100254197	protein GIGANTEA
31	100254521	probable glutathione S-transferase
32	100254890	ultraviolet-B receptor UVR8
33	100255072	OVARIAN TUMOR DOMAIN-containing deubiquitinating enzyme 12
34	100255485	galactinol synthase 1
35	100255655	F-box protein SKIP23-like
36	100255660	uncharacterized LOC100255660
37	100257397	co-chaperone protein p23-1
38	100258175	uncharacterized LOC100258175
39	100258274	probable plastid-lipid-associated protein 4, chloroplastic
40	100258986	peptidyl-prolyl cis-trans isomerase CYP37, chloroplastic
41	100259138	protein disulfide-isomerase
42	100259634	histone acetyltransferase MCC1
43	100261774	uncharacterized LOC100261774
44	100262037	putative pentatricopeptide repeat-containing protein At3g23330
45	100262887	chromatin modification-related protein EAF7
46	100266654	peptidyl-prolyl cis-trans isomerase FKBP62
47	100266884	uncharacterized LOC100266884
48	100267648	endoplasmin homolog
49	100852521	carnosine N-methyltransferase
50	100853585	pentatricopeptide repeat-containing protein At4g17616
51	100853980	putative disease resistance RPP13-like protein 1
52	104878259	peptidyl-prolyl cis-trans isomerase FKBP65
53	104880050	uncharacterized LOC104880050
54	104880245	uncharacterized LOC104880245
55	104881877	uncharacterized LOC104881877
56	novel.547	PF16594: Putative AtpZ or ATP-synthase-associated

Fig. S9 Gene Ontology analyses of key genes within *L. theobromae* F−red module that are correlated with grapevine G−blue module and temperature.

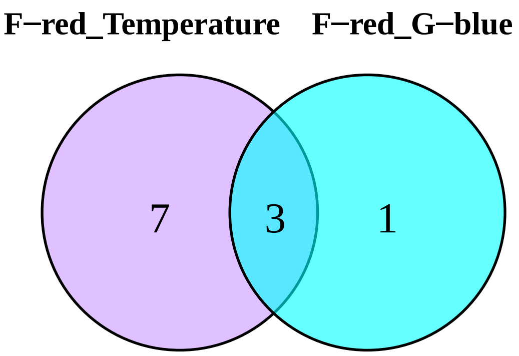

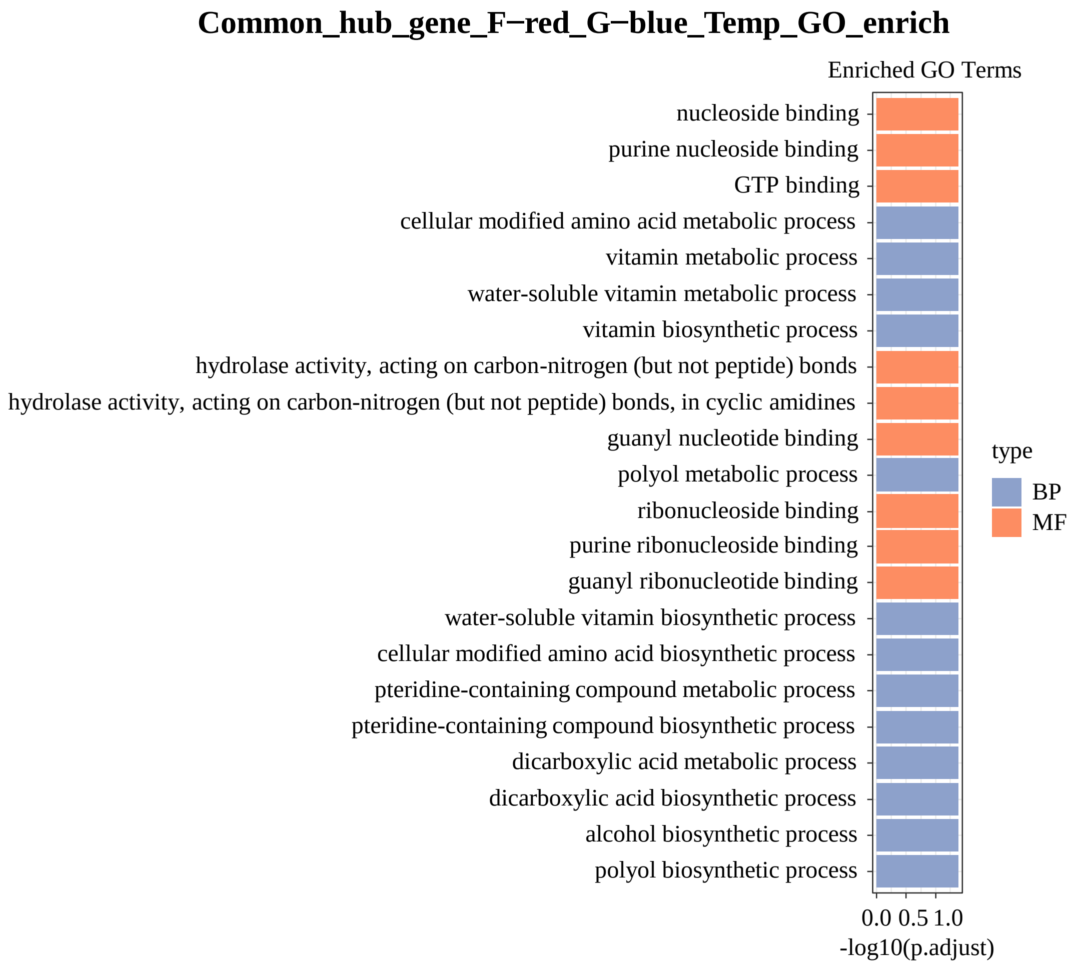

Common_hub_gene_F−red_G−blue_Temp_anno

	gene	description
1	g5330	GTP cyclohydrolase I
2	g9404	Glutathione S-transferase
3	g9410	NA

Fig. S10 Gene Ontology analyses of key genes within grapevine G−blue module that are correlated with *L. theobromae* F−brown module and temperature.

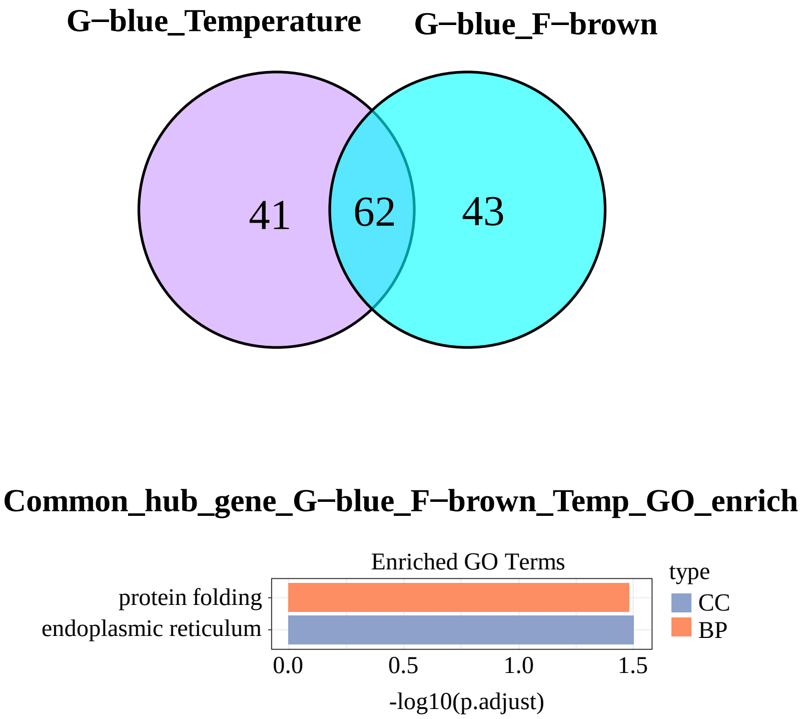

Common_hub_gene_G−blue_F−brown_Temp_anno

	gene	description
1	100241091	nudix hydrolase 14, chloroplastic-like
2	100241158	60S ribosomal protein L32-1
3	100241169	putative ALA-interacting subunit 2
4	100241270	putative pentatricopeptide repeat-containing protein At1g09680
5	100242205	protein POLLEN DEFECTIVE IN GUIDANCE 1
6	100242699	cysteine-rich receptor-like protein kinase 10
7	100243115	protein translation factor SUI1 homolog 1
8	100243120	protein disulfide isomerase-like 2-3
9	100244534	charged multivesicular body protein 5
10	100244643	14-3-3-like protein B
11	100245010	gibberellin 2-beta-dioxygenase 1
12	100246124	probable pectate lyase 12-like
13	100247016	uncharacterized protein At1g04910
14	100247365	GTP-binding nuclear protein Ran-3
15	100248135	calcyclin-binding protein
16	100248504	PRA1 family protein E
17	100248687	2-hydroxyisoflavanone dehydratase
18	100248914	chaperone protein DnaJ
19	100249097	peptidyl-prolyl cis-trans isomerase FKBP62
20	100249249	multiprotein-bridging factor 1c
21	100249262	uncharacterized LOC100249262
22	100249761	uncharacterized protein YwbO
23	100250042	non-specific lipid-transfer protein-like
24	100250368	FAD synthase
25	100250629	selenoprotein K-like
26	100251534	probable inactive ATP-dependent zinc metalloprotease FTSHI 2, chloroplastic
27	100251783	transcription repressor MYB6
28	100252105	dnaJ protein ERDJ3B
29	100252468	bifunctional purple acid phosphatase 26
30	100252886	purple acid phosphatase 23
31	100253622	multiple inositol polyphosphate phosphatase 1
32	100253897	vesicle-associated protein 1-3
33	100254197	protein GIGANTEA
34	100254521	probable glutathione S-transferase
35	100254890	ultraviolet-B receptor UVR8
36	100255485	galactinol synthase 1
37	100255655	F-box protein SKIP23-like
38	100255660	uncharacterized LOC100255660
39	100255990	probable sugar phosphate/phosphate translocator At4g32390
40	100258175	uncharacterized LOC100258175
41	100258274	probable plastid-lipid-associated protein 4, chloroplastic
42	100258986	peptidyl-prolyl cis-trans isomerase CYP37, chloroplastic
43	100259138	protein disulfide-isomerase
44	100259634	histone acetyltransferase MCC1
45	100259801	tetratricopeptide repeat protein SKI3
46	100260651	purple acid phosphatase
47	100261742	fanconi-associated nuclease 1 homolog
48	100261774	uncharacterized LOC100261774
49	100261821	uncharacterized LOC100261821
50	100262037	putative pentatricopeptide repeat-containing protein At3g23330
51	100262887	chromatin modification-related protein EAF7
52	100263126	DNA polymerase zeta catalytic subunit
53	100266409	mediator of RNA polymerase II transcription subunit 17
54	100266654	peptidyl-prolyl cis-trans isomerase FKBP62
55	100266884	uncharacterized LOC100266884
56	100267648	endoplasmin homolog
57	100853585	pentatricopeptide repeat-containing protein At4g17616
58	100853980	putative disease resistance RPP13-like protein 1
59	100854999	multiprotein-bridging factor 1a
60	104880245	uncharacterized LOC104880245
61	104880500	uncharacterized LOC104880500
62	104881877	uncharacterized LOC104881877

Fig. S11 Gene Ontology analyses of key genes within *L. theobromae* F−brown module that are correlated with grapevine G−blue module and temperature.

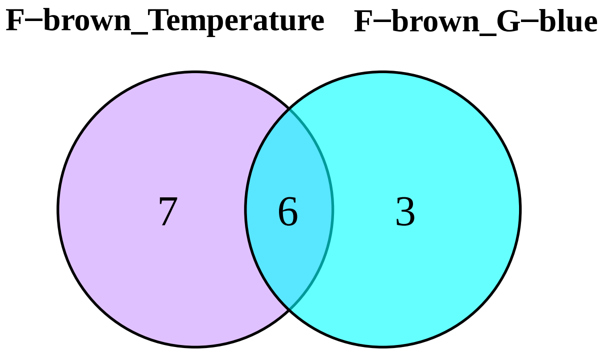

Common_hub_gene_F−brown_G−blue_ Temp_GO_enrich

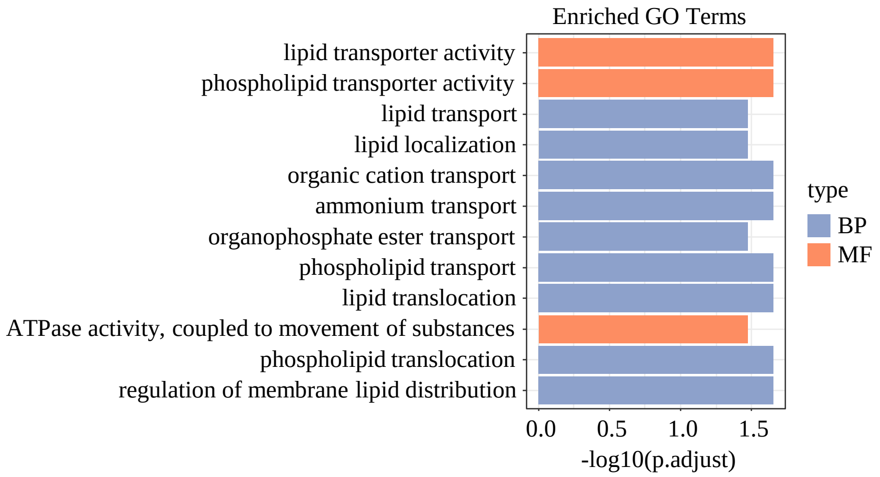

Common_hub_gene_F−brown_G−blue_Temp_anno

	gene	description
1	g11115	Rta1 domain protein
2	g12383	ABC transporter protein
3	g2437	Nudix domain protein
4	g2480	NADH:flavin oxidoreductase NADH oxidase
5	g7286	Phenylacetyl-ligase
6	g7634	Catechol O-methyltransferase

Fig. S12 Gene Ontology analyses of key genes within grapevine G−salmon module that are correlated with *L. theobromae* F−red module and temperature.

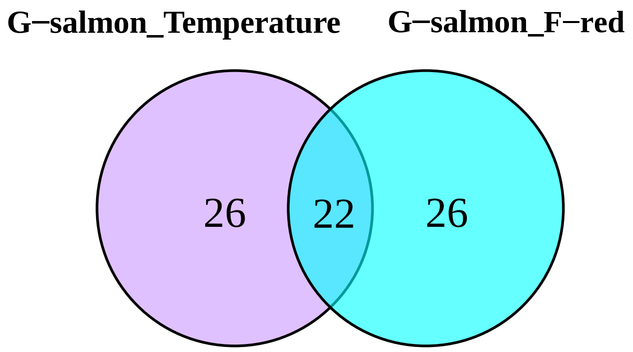

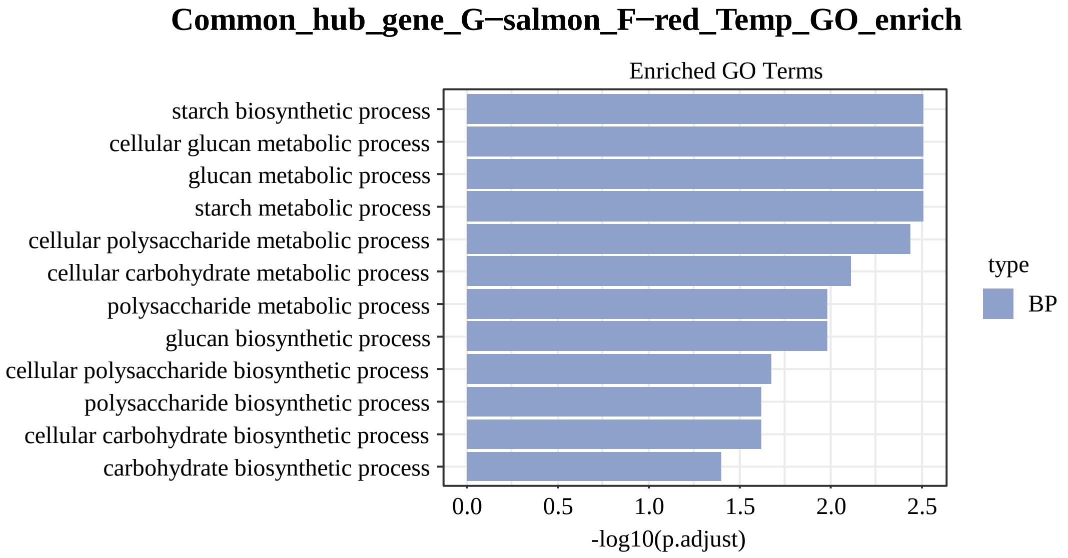

Common_hub_gene_G−salmon_F−red_Temp_anno

	gene	description
1	100241586	protein ALTERED XYLOGLUCAN 4
2	100241983	glucose-1-phosphate adenylyltransferase large subunit 1
3	100245665	transcription factor SPATULA
4	100247766	putative protein FAR1-RELATED SEQUENCE 10
5	100248070	uncharacterized LOC100248070
6	100250417	starch synthase 1, chloroplastic/amyloplastic
7	100250529	chaperone protein DnaJ
8	100252129	delta(12)-fatty-acid desaturase FAD2
9	100252368	filament-like plant protein
10	100254980	probable amino acid permease 7
11	100256357	probable tyrosine-protein phosphatase At1g05000
12	100256489	uncharacterized LOC100256489
13	100256713	(6-4)DNA photolyase
14	100257229	fatty acid desaturase 4, chloroplastic
15	100259022	sodium/hydrogen exchanger 2
16	100259502	transcription factor MYB24
17	100261788	DUF21 domain-containing protein At2g14520
18	100262687	uncharacterized LOC100262687
19	100266070	signal peptidase complex-like protein DTM1
20	100267134	uncharacterized methyltransferase At2g41040, chloroplastic
21	100855194	uncharacterized LOC100855194
22	novel.763	-

Fig. S13 Gene Ontology analyses of key genes within *L. theobromae* F−red module that are correlated with grapevine G−salmon module and temperature.

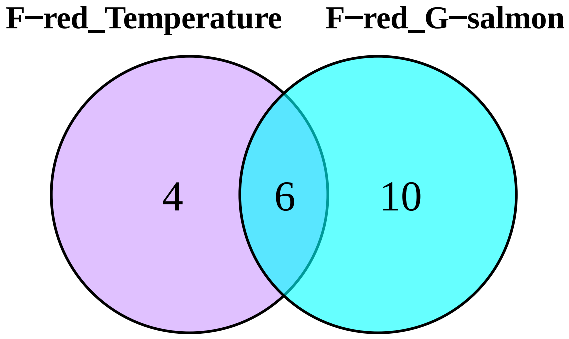

Common_hub_gene_F−red_G−salmon_Temp_anno

	gene	description
1	g10224	AAA family ATPAse
2	g2681	Polyketide synthase
3	g2686	Siderophore transporter, RhtX/FptX family
4	g3098	Cross-pathway control protein 1
5	g5330	GTP cyclohydrolase I
6	g9404	Glutathione S-transferase

Fig. S14 Gene Ontology analyses of key genes within grapevine G−salmon module that are correlated with *L. theobromae* F−brown and temperature.

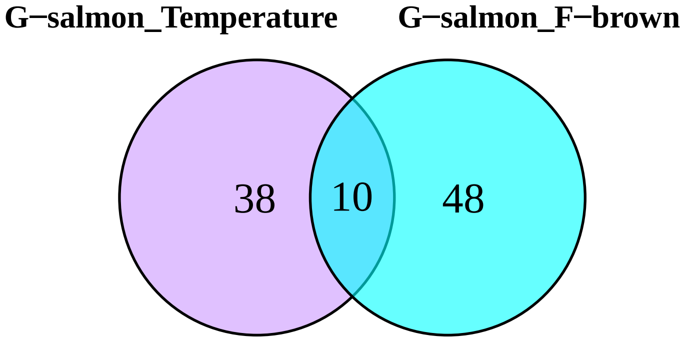

Common_hub_gene_G−salmon_F−brown_Temp_GO_enrich

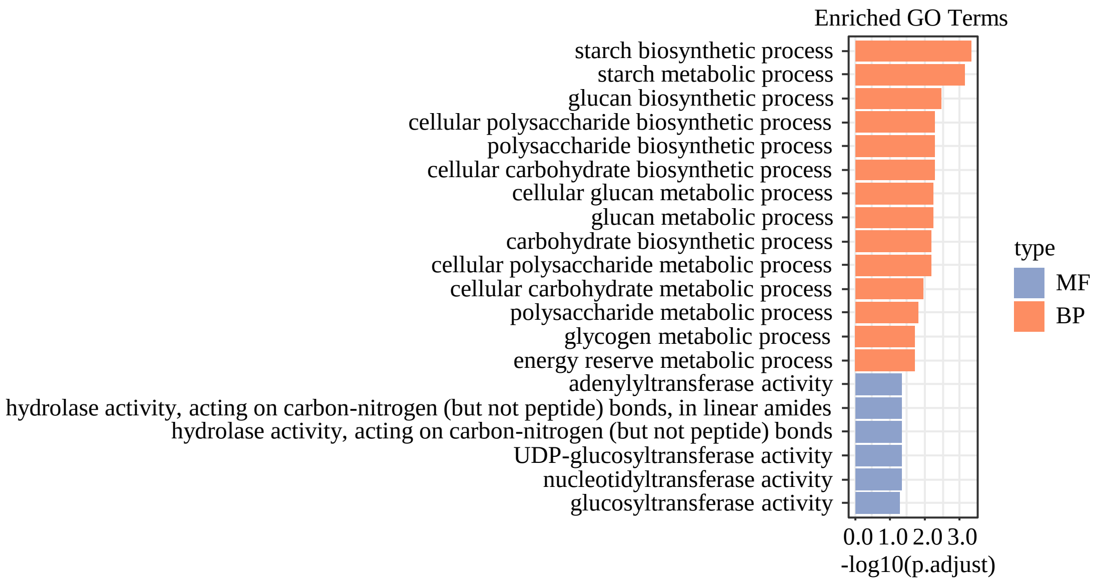

Common_hub_gene_G−salmon_F−brown_Temp_anno

	gene	description
1	100241983	glucose-1-phosphate adenylyltransferase large subunit 1
2	100245665	transcription factor SPATULA
3	100249424	palmitoyl-protein thioesterase 1
4	100250417	starch synthase 1, chloroplastic/amyloplastic
5	100252129	delta(12)-fatty-acid desaturase FAD2
6	100256713	(6-4)DNA photolyase
7	100257526	aminoacylase-1
8	100265331	probable bifunctional TENA-E protein
9	100266070	signal peptidase complex-like protein DTM1
10	100855194	uncharacterized LOC100855194

Fig. S15 Gene Ontology analyses of key genes within *L. theobromae* F−brown module that are correlated with grapevine G−salmon module and temperature.

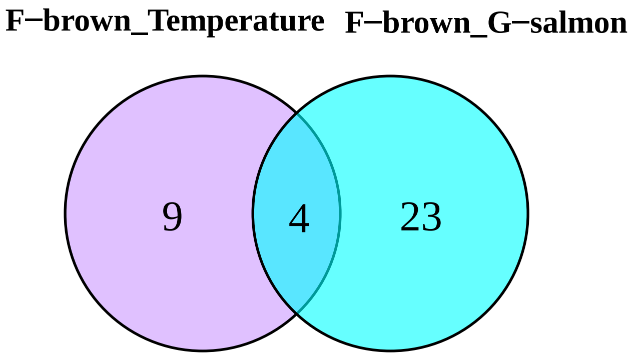

Common_hub_gene_F−brown_G−salmon_Temp_GO_enrich

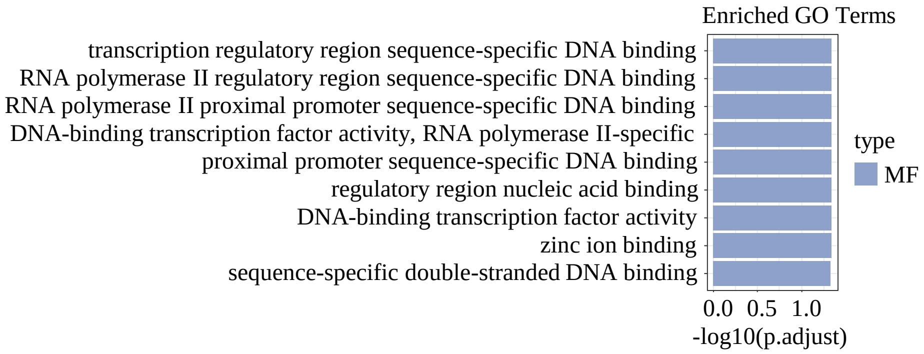

Common_hub_ gene_F−brown_G−salmon _Temp_anno

	gene	description
1	g2437	Nudix domain protein
2	g5563	Fungal specific transcription factor domain
3	g7069	ATPAse AAA+ type core protein
4	g7634	Catechol O-methyltransferase

Fig. S16 Gene Ontology analyses of key genes within grapevine G−salmon module that are correlated with *L. theobromae* F−darkred module and temperature

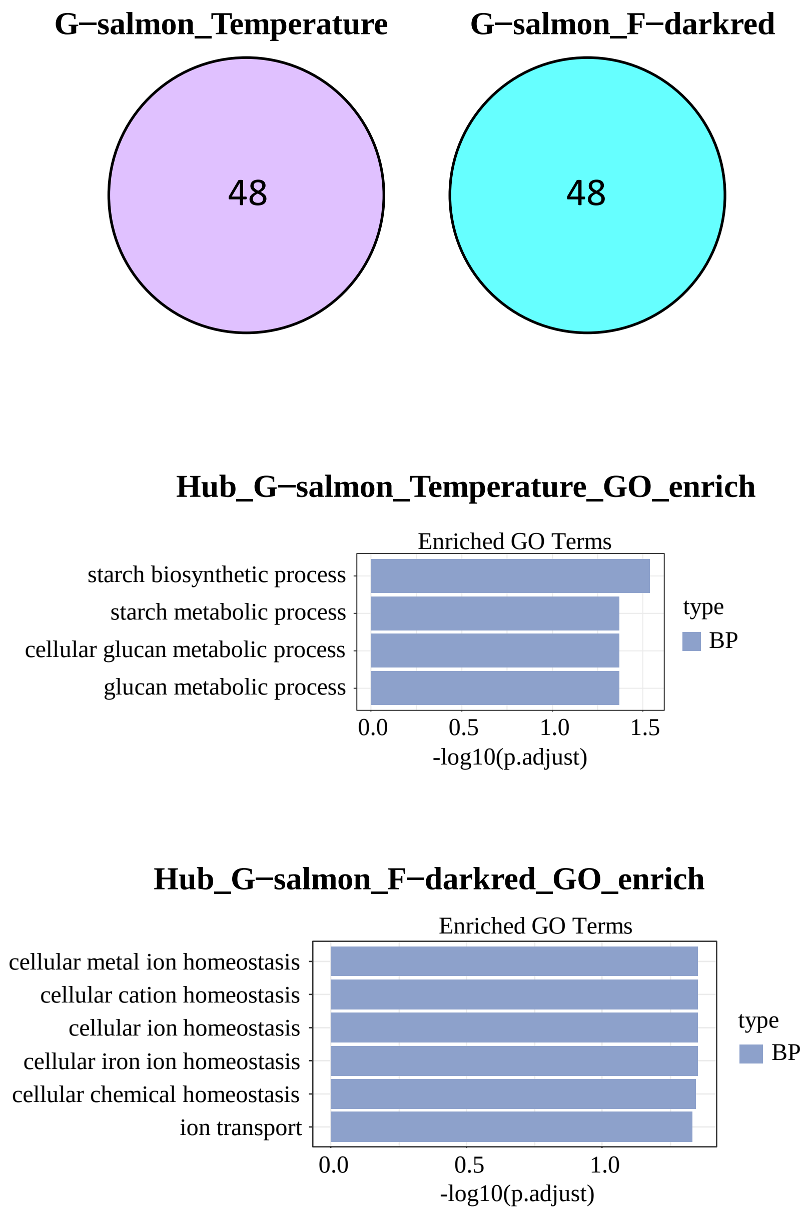

Fig. S17 Gene Ontology analyses of key genes within *L. theobromae* F−darkred module that are correlated with grapevine G−salmon module and temperature.

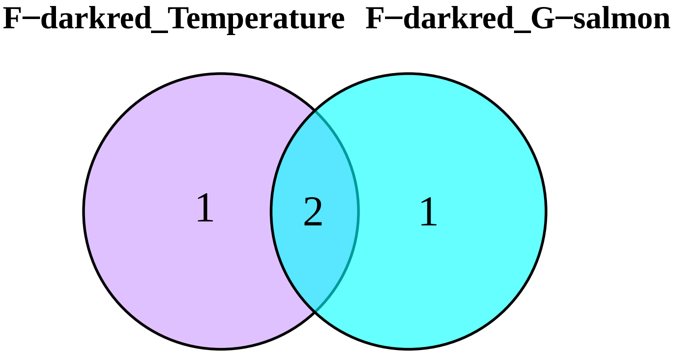

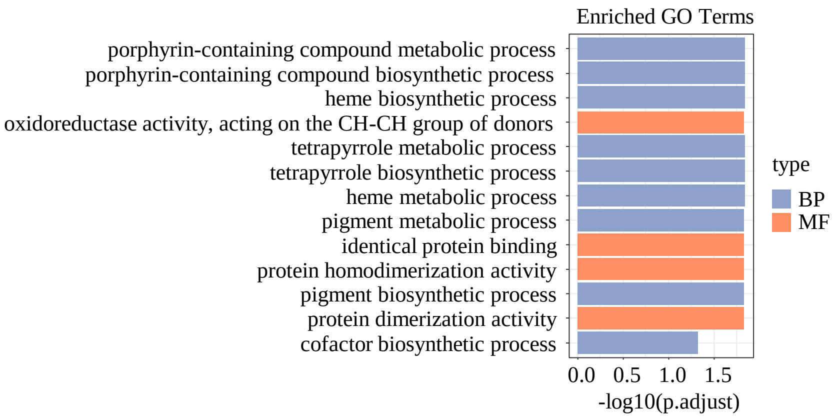

Common_hub_gene_F−darkred_G−salmon_Temp_anno

	gene	description
1	g6235	NA
2	g6412	Coproporphyrinogen III oxidase protein

Fig. S18 Gene Ontology analyses of key genes within grapevine G−green module that are correlated with *L. theobromae* F−brown module and temperature.

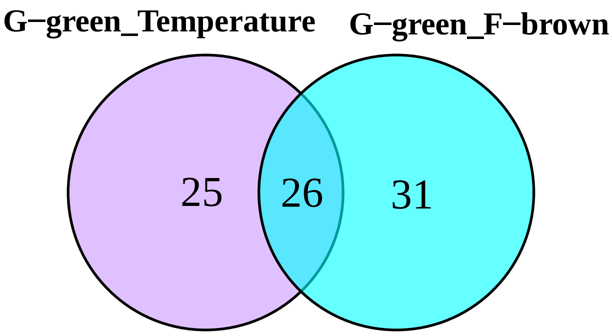

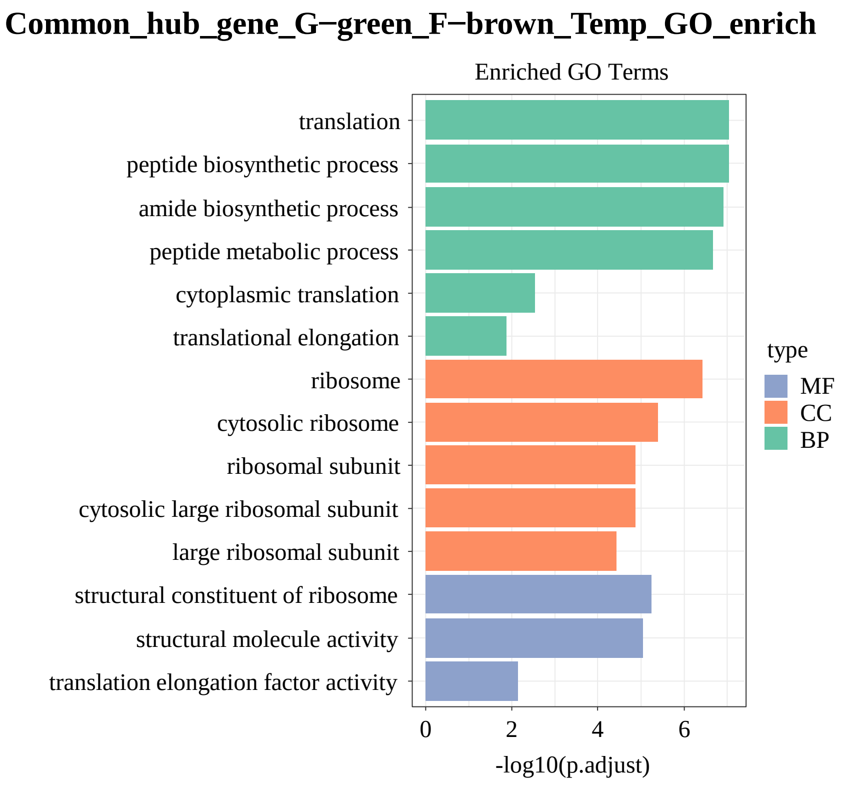

Common_hub_gene_G−green_F−brown_Temp_anno

	gene	description
1	100232932	peroxisomal (S)-2-hydroxy-acid oxidase GLO1
2	100241466	transcription factor bHLH106
3	100241564	nudix hydrolase 3
4	100242242	60S ribosomal protein L26-1
5	100244972	CBL-interacting protein kinase 11
6	100245662	3 beta-hydroxysteroid-dehydrogenase/decarboxylase
7	100247214	60S ribosomal protein L8
8	100247371	60S ribosomal protein L37-3
9	100247446	vacuolar iron transporter 1
10	100248037	40S ribosomal protein S27-2
11	100248155	uncharacterized LOC100248155
12	100248907	E3 ubiquitin ligase BIG BROTHER-related
13	100251464	60S ribosomal protein L11-1
14	100251538	-
15	100252536	40S ribosomal protein S7
16	100253421	CRS2-associated factor 2, chloroplastic
17	100253706	-
18	100255902	60S ribosomal protein L7a-1
19	100257178	40S ribosomal protein S8
20	100260533	60S ribosomal protein L3
21	100261522	elongation factor 1-beta 1
22	100262686	40S ribosomal protein S4-3
23	100266357	probable protein arginine N-methyltransferase 1
24	100852874	eukaryotic translation initiation factor 5A-2
25	100855039	60S ribosomal protein L26-1
26	109121414	AB hydrolase superfamily protein YfhM-like

Fig. S19 Gene Ontology analyses of key genes within *L. theobromae* F−brown module that are correlated with grapevine G−green module and temperature.

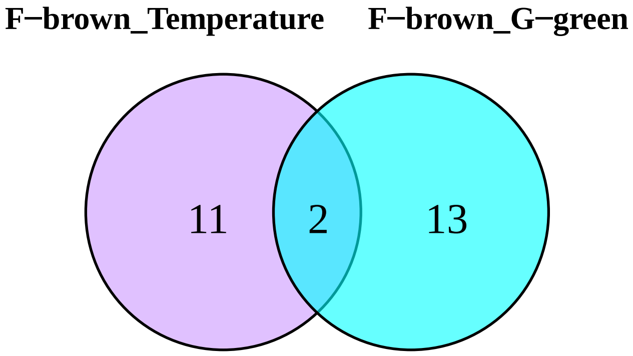

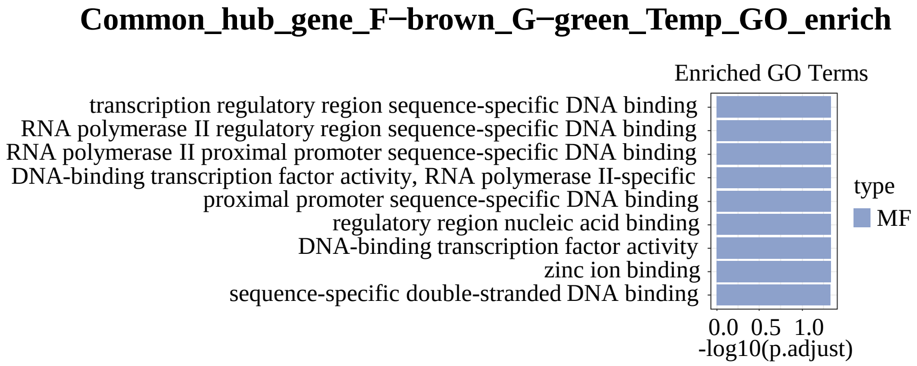

Common_hub_gene_F−brown_G−green_Temp_anno

	gene	description
1	g4867	NA
2	g5563	Fungal specific transcription factor domain

Fig. S20 Gene Ontology analyses of key genes within grapevine G−green module that are correlated with *L. theobromae* F−darkred module and temperature.

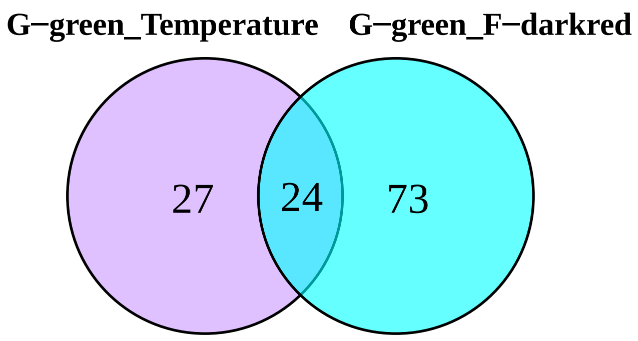

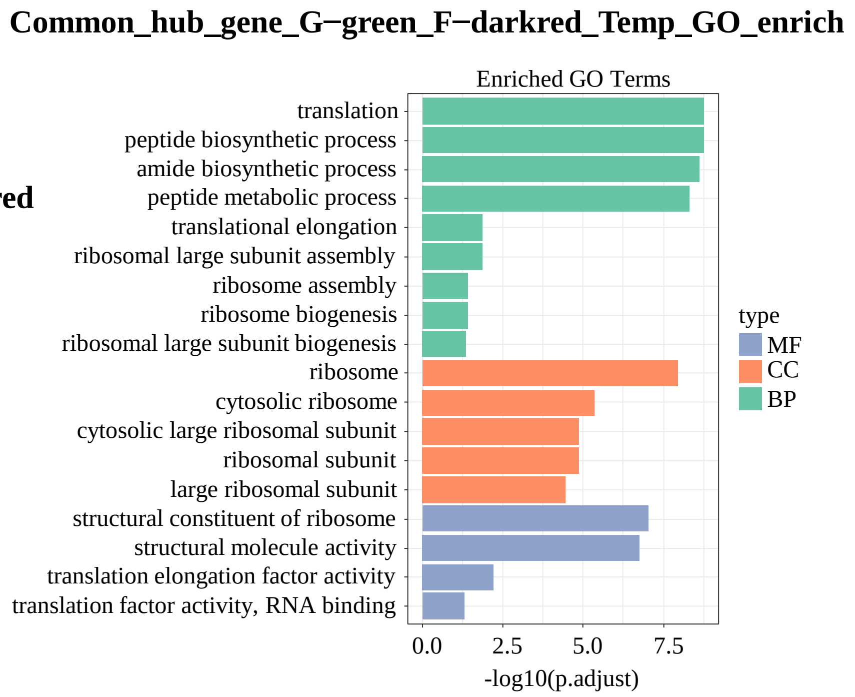

Common_hub_gene_ G−green_F−darkred _Temp_anno

	gene	gene_description
1	100244384	60S ribosomal protein L12-like
2	100248037	40S ribosomal protein S27-2
3	100248134	ABC transporter G family member 29
4	100248907	E3 ubiquitin ligase BIG BROTHER-related
5	100249575	T-complex protein 1 subunit alpha
6	100251414	cytochrome c oxidase assembly protein COX15
7	100251464	60S ribosomal protein L11-1
8	100251538	-
9	100251826	60S ribosomal protein L4
10	100252524	elongation factor 1-delta
11	100252536	40S ribosomal protein S7
12	100254719	60S ribosomal protein L23a
13	100255682	T-complex protein 1 subunit epsilon
14	100257178	40S ribosomal protein S8
15	100260208	60S ribosomal protein L14-1
16	100260533	60S ribosomal protein L3
17	100261522	elongation factor 1-beta 1
18	100261817	60S ribosomal protein L28-2
19	100262608	60S ribosomal protein L17-2
20	100262686	40S ribosomal protein S4-3
21	100265608	uncharacterized protein DDB_G0288133
22	100266487	60S ribosomal protein L10a-1
23	100852874	eukaryotic translation initiation factor 5A-2
24	109121414	AB hydrolase superfamily protein YfhM-like

Fig. S21 Gene Ontology analyses of key genes within *L. theobromae* F−darkred module that are correlated with grapevine G−green module and temperature.

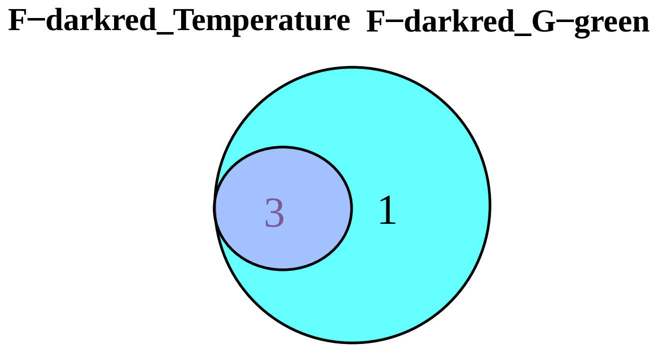

Common_hub_gene_F−darkred_G−green_Temp_GO_enrich

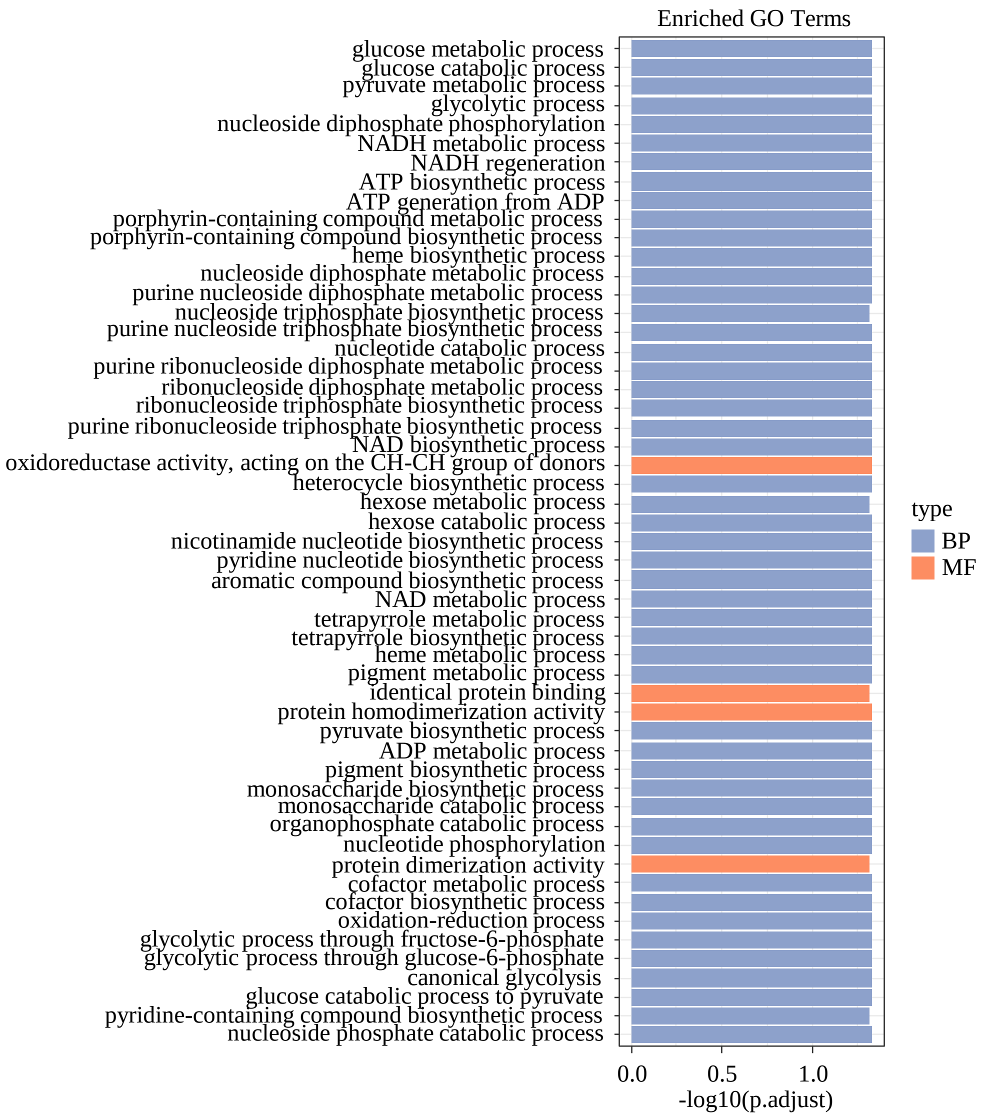

Common_hub_gene_F−darkred_G−green_Temp_anno

	gene	description
1	g3337	Phosphoglycerate kinase
2	g6235	NA
3	g6412	Coproporphyrinogen III oxidase protein
