# Supplementary material for: Dual RNA-Seq Reveals Temperature-Mediated Gene Reprogramming and Molecular Crosstalk between Grapevine and Lasiodiplodia theobromae
Source: J Fungi (Basel). 2023 Dec 14;9(12):1197. doi: 10.3390/jof9121197 (PMC10745131; doi:10.3390/jof9121197)
Supplement: Supplementary file 1 [file jof-09-01197-s001.zip › Table S1.pdf]

Supplementary Table 1. Primers used in the study.

Primer name	Sequence （5’-3’）	Use of primer
100268095-f	CAAGTGAAGGTAACCGTTCT	Used for RT-qPCR assay
100268095-r	CTGCTTCTGTTCTGGTATTGA	
100250417-f	GGAATTAAGCCTGCCCATTAG	Used for RT-qPCR assay
100250417-r	CCATCACTCTCATCCACTCTT	
100243120-f	GAGTGGAAGAAGGCTGCTAAT	Used for RT-qPCR assay
100243120-r	GCACCGAACACCAAGATAGT	
100252105-f	GAGAACCTGGAGACCTGAAGT	Used for RT-qPCR assay
100252105-r	AAGCCAACAAGAGCCTGAAC	
100246301-f	TGTTCTTCGTCTCGCTTTCT	Used for RT-qPCR assay
100246301-r	GTCGTTCATCCATCCACCTAT	
100266654-f	GGACTTTGCTTGATGGAACTC	Used for RT-qPCR assay
100266654-r	GGAGGAATGGTTGTTGAAGAC	
VvActin-f	CTACTCCTTCACCACCACAG	Used for the internal control of RT-qPCR detection
VvActin-r	TCCATCAGGCAACTCATAGC	
g3337-f	GACGGCAAGAGGAACGAGAAG	Used for RT-qPCR assay
g3337-r	CCAACGCAGTCGCTAGTGAA	
g2480-f	CGCAGAACGGATGAGTATGG	Used for RT-qPCR assay
g2480-r	CGACCGAGTTGAGCTTGATT	
g6412-f	ACGCCATCCACTTCCACAAG	Used for RT-qPCR assay
g6412-r	TCCGTCTCGTCCAAGTCATCA	

g12383-f	CGTGAATGGCGAGGGTTCTG	Used for RT-qPCR assay
g12383-r	GGTTATGGCGTGCTGGTTGAT	
g2686-f	TCATTGTGACGGATCTGGTT	Used for RT-qPCR assay
g2686-r	ATGAAGATGACGACGAAGGT	
g7069-f	GACTACGCAACGGAGAAGAG	Used for RT-qPCR assay
g7069-r	GGAAGAAGTGGTCGTCTGAG	
LtActin-f	CCAAGTCCAACCGTGAGAAG	Used for the internal control of RT-qPCR detection
LtActin-r	GAAGCGTACAGCGACAGAAC	
