# Supplementary material for: Dual RNA-Seq Reveals Temperature-Mediated Gene Reprogramming and Molecular Crosstalk between Grapevine and Lasiodiplodia theobromae
Source: J Fungi (Basel). 2023 Dec 14;9(12):1197. doi: 10.3390/jof9121197 (PMC10745131; doi:10.3390/jof9121197)
Supplement: Supplementary file 1 [file jof-09-01197-s001.zip › Table S2.pdf]

Supplementary Table 2. Summary results of the sequencing reads of grapevine samples infected by *L. theobromae*.

Smaples	Raw_reads	Clean_reads	map_to_grape	grape_map_ratio	map_to_fungi	fungi_map_ratio
Y24T25_1	65037081	63562580	51199564	80.55%	1201724	1.89%
Y24T25_2	61404023	59834598	48671764	81.34%	903614	1.51%
Y24T25_3	61742918	60136126	48398502	80.48%	923256	1.54%
Y24T30_1	65080539	63740330	52541714	82.43%	274652	0.43%
Y24T30_2	54650481	53502304	43613938	81.52%	229386	0.43%
Y24T30_3	59616046	58401020	47556218	81.43%	472232	0.81%
Y24T35_1	51620779	50116030	39933094	79.68%	636978	1.27%
Y24T35_2	57714664	56724110	46278792	81.59%	293312	0.52%
Y24T35_3	57752708	56250564	45949744	81.69%	302800	0.54%
Y48T25_1	51513853	50469300	40296506	79.84%	816674	1.62%
Y48T25_2	53178005	52069812	42338598	81.31%	520078	1.00%
Y48T25_3	54907292	53734174	42877582	79.80%	862380	1.60%
Y48T30_1	44571670	43527982	35993852	82.69%	106510	0.24%
Y48T30_2	53195953	51994096	42435594	81.62%	114634	0.22%
Y48T30_3	46917202	45389888	37253580	82.07%	155370	0.34%
Y48T35_1	59209021	57430506	46779302	81.45%	136790	0.24%
Y48T35_2	59935500	58403616	46385984	79.42%	713802	1.22%
Y48T35_3	55503076	54118246	44706080	82.61%	113920	0.21%
Y72T25_1	56189138	54793648	45012732	82.15%	77438	0.14%
Y72T25_2	59666933	58392448	48298830	82.71%	145198	0.25%
Y72T25_3	49647814	48569196	39900464	82.15%	335804	0.69%
Y72T30_1	56028685	54556356	44145726	80.92%	438794	0.80%
Y72T30_2	59824543	58316216	46988242	80.57%	520812	0.89%
Y72T30_3	52434644	51259420	42316632	82.55%	238038	0.46%
Y72T35_1	57311036	56146454	45385732	80.83%	154380	0.27%
Y72T35_2	53702323	52463016	42074454	80.20%	502222	0.96%
Y72T35_3	56963238	55567494	44916006	80.83%	145368	0.26%
