# Supplementary material for: Dual RNA-Seq Reveals Temperature-Mediated Gene Reprogramming and Molecular Crosstalk between Grapevine and Lasiodiplodia theobromae
Source: J Fungi (Basel). 2023 Dec 14;9(12):1197. doi: 10.3390/jof9121197 (PMC10745131; doi:10.3390/jof9121197)
Supplement: Supplementary file 1 [file jof-09-01197-s001.zip › Table S3.pdf]

Supplementary Table 3. List of grapevine temperature-correlated genes found in correlated modules between grapevine and *L. theobromae*.

	Grapevine _module	*L. theobromae* _module	Gene	Description
1	G-pink	F-brown	100232889	ripening-related bZIP protein-like
2	G-pink	F-brown	100233076	glucan endo-1, 3-beta-glucosidase
3	G-pink	F-brown	100244508	adagio protein 1
4	G-pink	F-brown	100245170	O-fucosyltransferase 15
5	G-pink	F-brown	100246781	cytochrome P450 736A117
6	G-pink	F-brown	100247186	protein detoxification 27
7	G-pink	F-brown	100247368	uncharacterized LOC100247368
8	G-pink	F-brown	100248690	cold-regulated 413 plasma membrane protein 2
9	G-pink	F-brown	100251651	uncharacterized LOC100251651
10	G-pink	F-brown	100253180	CBL-interacting protein kinase 04
11	G-pink	F-brown	100260954	protein MEI2-like 4
12	G-pink	F-brown	100261257	cysteine proteinase COT44
13	G-pink	F-brown	100262654	sec1 family domain-containing protein MIP3
14	G-pink	F-brown	100264030	phosphatidylinositol 4-kinase gamma 3
15	G-pink	F-brown	100264466	uncharacterized LOC100264466
16	G-pink	F-brown	100265388	putative BPI/LBP family protein At1g04970
17	G-pink	F-brown	100265471	probable xyloglucan endotransglucosylase/hydrolase protein 32
18	G-pink	F-brown	100266331	aldehyde dehydrogenase family 3 member H1
19	G-pink	F-brown	100266362	paramyosin
20	G-pink	F-brown	100266572	glycine-rich domain-containing protein 2
21	G-pink	F-brown	100268095	tocopherol cyclase, chloroplastic
22	G-pink	F-brown	novel.408	-
1	G-pink	F-red	100232889	ripening-related bZIP protein-like
2	G-pink	F-red	100244508	adagio protein 1
3	G-pink	F-red	100246781	cytochrome P450 71A3
4	G-pink	F-red	100247186	protein detoxification 27
5	G-pink	F-red	100247368	uncharacterized LOC100247368
6	G-pink	F-red	100248696	autophagy-related protein 8i
7	G-pink	F-red	100253180	CBL-interacting protein kinase 04
8	G-pink	F-red	100256125	uncharacterized LOC100256125
9	G-pink	F-red	100260954	protein MEI2-like 4
10	G-pink	F-red	100261030	protein MEI2-like 4

11	G-pink	F-red	100262863	profilin-1
12	G-pink	F-red	100262931	uncharacterized LOC100262931
13	G-pink	F-red	100264190	WD repeat-containing protein 44
14	G-pink	F-red	100264466	uncharacterized LOC100264466
15	G-pink	F-red	100265471	probable xyloglucan endotransglucosylase/hydrolase protein 32
16	G-pink	F-red	100265815	vacuolar amino acid transporter 1
17	G-pink	F-red	100266050	GABA transporter 1
18	G-pink	F-red	100266130	box C/D snoRNA protein 1
19	G-pink	F-red	100266362	paramyosin
20	G-pink	F-red	100266572	glycine-rich domain-containing protein 2
21	G-pink	F-red	104877310	beta-glucosidase 18
22	G-pink	F-red	novel.408	-
1	G-pink	F-darkred	100244508	adagio protein 1
2	G-pink	F-darkred	100246301	probable inositol transporter 2
3	G-pink	F-darkred	100247561	deoxyhypusine synthase
4	G-pink	F-darkred	100248377	omega-6 fatty acid desaturase, chloroplastic
5	G-pink	F-darkred	100249560	AT-hook motif nuclear-localized protein 5
6	G-pink	F-darkred	100250069	protein ENHANCED DISEASE RESISTANCE 2-like
7	G-pink	F-darkred	100251651	uncharacterized LOC100251651
8	G-pink	F-darkred	100254964	cysteine-rich and transmembrane domain-containing protein A
9	G-pink	F-darkred	100255560	LRR receptor-like serine/threonine-protein kinase GSO2
10	G-pink	F-darkred	100256125	uncharacterized LOC100256125
11	G-pink	F-darkred	100259117	uncharacterized LOC100259117
12	G-pink	F-darkred	100260954	protein MEI2-like 4
13	G-pink	F-darkred	100261030	protein MEI2-like 4
14	G-pink	F-darkred	100261257	cysteine proteinase COT44
15	G-pink	F-darkred	100263828	RINT1-like protein MAG2
16	G-pink	F-darkred	100264466	uncharacterized LOC100264466
17	G-pink	F-darkred	100265388	putative BPI/LBP family protein At1g04970
18	G-pink	F-darkred	100265815	vacuolar amino acid transporter 1
19	G-pink	F-darkred	100266362	paramyosin
20	G-pink	F-darkred	100268095	tocopherol cyclase, chloroplastic
1	G-blue	F-red	100241091	nudix hydrolase 14, chloroplastic-like
2	G-blue	F-red	100241158	60S ribosomal protein L32-1
3	G-blue	F-red	100241169	putative ALA-interacting subunit 2
4	G-blue	F-red	100241270	putative pentatricopeptide repeat-containing protein At1g09680
5	G-blue	F-red	100242205	protein POLLEN DEFECTIVE IN GUIDANCE 1
6	G-blue	F-red	100243115	protein translation factor SUI1 homolog 1

7	G-blue	F-red	100243120	protein disulfide isomerase-like 2-3
8	G-blue	F-red	100243248	transcription factor RF2b
9	G-blue	F-red	100244643	14-3-3-like protein B
10	G-blue	F-red	100246124	probable pectate lyase 12-like
11	G-blue	F-red	100246648	RNA-binding protein 42
12	G-blue	F-red	100247016	uncharacterized protein At1g04910
13	G-blue	F-red	100247365	GTP-binding nuclear protein Ran-3
14	G-blue	F-red	100248135	calcyclin-binding protein
15	G-blue	F-red	100248504	PRA1 family protein E
16	G-blue	F-red	100248687	2-hydroxyisoflavanone dehydratase
17	G-blue	F-red	100248914	chaperone protein DnaJ
18	G-blue	F-red	100249097	peptidyl-prolyl cis-trans isomerase FKBP62
19	G-blue	F-red	100249249	multiprotein-bridging factor 1c
20	G-blue	F-red	100249262	uncharacterized LOC100249262
21	G-blue	F-red	100249761	uncharacterized protein YwbO
22	G-blue	F-red	100250042	non-specific lipid-transfer protein-like
23	G-blue	F-red	100250368	FAD synthase
24	G-blue	F-red	100250629	selenoprotein K-like
25	G-blue	F-red	100251534	probable inactive ATP-dependent zinc metalloprotease FTSHI 2, chloroplastic
26	G-blue	F-red	100252105	dnaJ protein ERDJ3B
27	G-blue	F-red	100252468	bifunctional purple acid phosphatase 26
28	G-blue	F-red	100252886	purple acid phosphatase 23
29	G-blue	F-red	100253897	vesicle-associated protein 1-3
30	G-blue	F-red	100254197	protein GIGANTEA
31	G-blue	F-red	100254521	probable glutathione S-transferase
32	G-blue	F-red	100254890	ultraviolet-B receptor UVR8
33	G-blue	F-red	100255072	OTU domain-containing protein DDB_G0284757
34	G-blue	F-red	100255485	galactinol synthase 1
35	G-blue	F-red	100255655	F-box protein SKIP23-like
36	G-blue	F-red	100255660	uncharacterized LOC100255660
37	G-blue	F-red	100257397	uncharacterized protein OsI_027940
38	G-blue	F-red	100258175	uncharacterized LOC100258175
39	G-blue	F-red	100258274	probable plastid-lipid-associated protein 4, chloroplastic
40	G-blue	F-red	100258986	peptidyl-prolyl cis-trans isomerase CYP37, chloroplastic
41	G-blue	F-red	100259138	protein disulfide-isomerase
42	G-blue	F-red	100259634	histone acetyltransferase MCC1
43	G-blue	F-red	100261774	uncharacterized LOC100261774
44	G-blue	F-red	100262037	putative pentatricopeptide repeat-containing protein At3g23330

45	G-blue	F-red	100262887	chromatin modification-related protein EAF7
46	G-blue	F-red	100266654	peptidyl-prolyl cis-trans isomerase FKBP62
47	G-blue	F-red	100266884	uncharacterized LOC100266884
48	G-blue	F-red	100267648	endoplasmin homolog
49	G-blue	F-red	100852521	carnosine N-methyltransferase
50	G-blue	F-red	100853585	pentatricopeptide repeat-containing protein At4g17616
51	G-blue	F-red	100853980	putative disease resistance RPP13-like protein 1
52	G-blue	F-red	104878259	peptidyl-prolyl cis-trans isomerase FKBP65
53	G-blue	F-red	104880050	uncharacterized LOC104880050
54	G-blue	F-red	104880245	uncharacterized LOC104880245
55	G-blue	F-red	104881877	uncharacterized LOC104881877
56	G-blue	F-red	novel.547	PF16594: Putative AtpZ or ATP-synthase-associated
1	G-blue	F-brown	100241091	nudix hydrolase 14, chloroplastic-like
2	G-blue	F-brown	100241158	60S ribosomal protein L32-1
3	G-blue	F-brown	100241169	putative ALA-interacting subunit 2
4	G-blue	F-brown	100241270	putative pentatricopeptide repeat-containing protein At1g09680
5	G-blue	F-brown	100242205	protein POLLEN DEFECTIVE IN GUIDANCE 1
6	G-blue	F-brown	100242699	cysteine-rich receptor-like protein kinase 10
7	G-blue	F-brown	100243115	protein translation factor SUI1 homolog 1
8	G-blue	F-brown	100243120	protein disulfide isomerase-like 2-3
9	G-blue	F-brown	100244534	charged multivesicular body protein 5
10	G-blue	F-brown	100244643	14-3-3-like protein B
11	G-blue	F-brown	100245010	gibberellin 2-beta-dioxygenase 1
12	G-blue	F-brown	100246124	probable pectate lyase 12-like
13	G-blue	F-brown	100247016	uncharacterized protein At1g04910
14	G-blue	F-brown	100247365	GTP-binding nuclear protein Ran-3
15	G-blue	F-brown	100248135	calcyclin-binding protein
16	G-blue	F-brown	100248504	PRA1 family protein E
17	G-blue	F-brown	100248687	2-hydroxyisoflavanone dehydratase
18	G-blue	F-brown	100248914	chaperone protein DnaJ
19	G-blue	F-brown	100249097	peptidyl-prolyl cis-trans isomerase FKBP62
20	G-blue	F-brown	100249249	multiprotein-bridging factor 1c
21	G-blue	F-brown	100249262	uncharacterized LOC100249262
22	G-blue	F-brown	100249761	uncharacterized protein YwbO
23	G-blue	F-brown	100250042	non-specific lipid-transfer protein-like
24	G-blue	F-brown	100250368	FAD synthase
25	G-blue	F-brown	100250629	selenoprotein K-like

26	G-blue	F-brown	100251534	probable inactive ATP-dependent zinc metalloprotease FTSHI 2, chloroplastic
27	G-blue	F-brown	100251783	transcription repressor MYB6
28	G-blue	F-brown	100252105	dnaJ protein ERDJ3B
29	G-blue	F-brown	100252468	bifunctional purple acid phosphatase 26
30	G-blue	F-brown	100252886	purple acid phosphatase 23
31	G-blue	F-brown	100253622	multiple inositol polyphosphate phosphatase 1
32	G-blue	F-brown	100253897	vesicle-associated protein 1-3
33	G-blue	F-brown	100254197	protein GIGANTEA
34	G-blue	F-brown	100254521	probable glutathione S-transferase
35	G-blue	F-brown	100254890	ultraviolet-B receptor UVR8
36	G-blue	F-brown	100255485	galactinol synthase 1
37	G-blue	F-brown	100255655	F-box protein SKIP23-like
38	G-blue	F-brown	100255660	uncharacterized LOC100255660
39	G-blue	F-brown	100255990	probable sugar phosphate/phosphate translocator At4g32390
40	G-blue	F-brown	100258175	uncharacterized LOC100258175
41	G-blue	F-brown	100258274	probable plastid-lipid-associated protein 4, chloroplastic
42	G-blue	F-brown	100258986	peptidyl-prolyl cis-trans isomerase CYP37, chloroplastic
43	G-blue	F-brown	100259138	protein disulfide-isomerase
44	G-blue	F-brown	100259634	histone acetyltransferase MCC1
45	G-blue	F-brown	100259801	tetratricopeptide repeat protein SKI3
46	G-blue	F-brown	100260651	purple acid phosphatase
47	G-blue	F-brown	100261742	fanconi-associated nuclease 1 homolog
48	G-blue	F-brown	100261774	uncharacterized LOC100261774
49	G-blue	F-brown	100261821	uncharacterized LOC100261821
50	G-blue	F-brown	100262037	putative pentatricopeptide repeat-containing protein At3g23330
51	G-blue	F-brown	100262887	chromatin modification-related protein EAF7
52	G-blue	F-brown	100263126	DNA polymerase zeta catalytic subunit
53	G-blue	F-brown	100266409	mediator of RNA polymerase II transcription subunit 17
54	G-blue	F-brown	100266654	peptidyl-prolyl cis-trans isomerase FKBP62
55	G-blue	F-brown	100266884	uncharacterized LOC100266884
56	G-blue	F-brown	100267648	endoplasmin homolog
57	G-blue	F-brown	100853585	pentatricopeptide repeat-containing protein At4g17616
58	G-blue	F-brown	100853980	putative disease resistance RPP13-like protein 1
59	G-blue	F-brown	100854999	multiprotein-bridging factor 1a

60	G-blue	F-brown	104880245	uncharacterized LOC104880245
61	G-blue	F-brown	104880500	uncharacterized LOC104880500
62	G-blue	F-brown	104881877	uncharacterized LOC104881877
1	G-salmon	F-red	100241586	protein ALTERED XYLOGLUCAN 4
2	G-salmon	F-red	100241983	glucose-1-phosphate adenylyltransferase large subunit 1
3	G-salmon	F-red	100245665	transcription factor SPATULA
4	G-salmon	F-red	100247766	putative protein FAR1-RELATED SEQUENCE 10
5	G-salmon	F-red	100248070	uncharacterized LOC100248070
6	G-salmon	F-red	100250417	starch synthase 1, chloroplastic/amyloplastic
7	G-salmon	F-red	100250529	chaperone protein DnaJ
8	G-salmon	F-red	100252129	delta(12)-fatty-acid desaturase FAD2
9	G-salmon	F-red	100252368	filament-like plant protein
10	G-salmon	F-red	100254980	probable amino acid permease 7
11	G-salmon	F-red	100256357	probable tyrosine-protein phosphatase DSP4
12	G-salmon	F-red	100256489	uncharacterized LOC100256489
13	G-salmon	F-red	100256713	(6-4)DNA photolyase
14	G-salmon	F-red	100257229	fatty acid desaturase 4, chloroplastic
15	G-salmon	F-red	100259022	sodium/hydrogen exchanger 2
16	G-salmon	F-red	100259502	transcription factor MYB24
17	G-salmon	F-red	100261788	DUF21 domain-containing protein At2g14520
18	G-salmon	F-red	100262687	uncharacterized LOC100262687
19	G-salmon	F-red	100266070	signal peptidase complex-like protein DTM1
20	G-salmon	F-red	100267134	methyltransferase At2g41040, chloroplastic
21	G-salmon	F-red	100855194	uncharacterized LOC100855194
22	G-salmon	F-red	novel.763	-
1	G-salmon	F-brown	100241983	glucose-1-phosphate adenylyltransferase large subunit 1
2	G-salmon	F-brown	100245665	transcription factor SPATULA
3	G-salmon	F-brown	100249424	palmitoyl-protein thioesterase 1
4	G-salmon	F-brown	100250417	starch synthase 1, chloroplastic/amyloplastic
5	G-salmon	F-brown	100252129	delta(12)-fatty-acid desaturase FAD2
6	G-salmon	F-brown	100256713	(6-4)DNA photolyase
7	G-salmon	F-brown	100257526	aminoacylase-1
8	G-salmon	F-brown	100265331	probable bifunctional TENA-E protein
9	G-salmon	F-brown	100266070	signal peptidase complex-like protein DTM1
10	G-salmon	F-brown	100855194	uncharacterized LOC100855194
1	G-green	F-brown	100232932	peroxisomal (S)-2-hydroxy-acid oxidase GLO1
2	G-green	F-brown	100241466	transcription factor bHLH106
3	G-green	F-brown	100241564	nudix hydrolase 3
4	G-green	F-brown	100242242	60S ribosomal protein L26-1

5	G-green	F-brown	100244972	CBL-interacting protein kinase 11
6	G-green	F-brown	100245662	3 beta-hydroxysteroid-dehydrogenase/decarboxylase
7	G-green	F-brown	100247214	60S ribosomal protein L8
8	G-green	F-brown	100247371	60S ribosomal protein L37-3
9	G-green	F-brown	100247446	vacuolar iron transporter 1
10	G-green	F-brown	100248037	40S ribosomal protein S27-2
11	G-green	F-brown	100248155	uncharacterized LOC100248155
12	G-green	F-brown	100248907	E3 ubiquitin ligase BIG BROTHER-related
13	G-green	F-brown	100251464	60S ribosomal protein L11-1
14	G-green	F-brown	100251538	-
15	G-green	F-brown	100252536	40S ribosomal protein S7
16	G-green	F-brown	100253421	CRS2-associated factor 2 chloroplastic
17	G-green	F-brown	100253706	-
18	G-green	F-brown	100255902	60S ribosomal protein L7a-1
19	G-green	F-brown	100257178	40S ribosomal protein S8
20	G-green	F-brown	100260533	60S ribosomal protein L3
21	G-green	F-brown	100261522	elongation factor 1-beta 1
22	G-green	F-brown	100262686	40S ribosomal protein S4-3
23	G-green	F-brown	100266357	probable protein arginine N-methyltransferase 1
24	G-green	F-brown	100852874	eukaryotic translation initiation factor 5A-2
25	G-green	F-brown	100855039	60S ribosomal protein L26-1
26	G-green	F-brown	109121414	AB hydrolase superfamily protein YfhM-like
1	G-green	F-darkred	100244384	60S ribosomal protein L12-like
2	G-green	F-darkred	100248037	40S ribosomal protein S27-2
3	G-green	F-darkred	100248134	ABC transporter G family member 29
4	G-green	F-darkred	100248907	E3 ubiquitin ligase BIG BROTHER-related
5	G-green	F-darkred	100249575	T-complex protein 1 subunit alpha
6	G-green	F-darkred	100251414	cytochrome c oxidase assembly protein COX15
7	G-green	F-darkred	100251464	60S ribosomal protein L11-1
8	G-green	F-darkred	100251538	-
9	G-green	F-darkred	100251826	60S ribosomal protein L4
10	G-green	F-darkred	100252524	elongation factor 1-delta
11	G-green	F-darkred	100252536	40S ribosomal protein S7
12	G-green	F-darkred	100254719	60S ribosomal protein L23a
13	G-green	F-darkred	100255682	T-complex protein 1 subunit epsilon
14	G-green	F-darkred	100257178	40S ribosomal protein S8
15	G-green	F-darkred	100260208	60S ribosomal protein L14-1
16	G-green	F-darkred	100260533	60S ribosomal protein L3
17	G-green	F-darkred	100261522	elongation factor 1-beta 1
18	G-green	F-darkred	100261817	60S ribosomal protein L28-2

19	G-green	F-darkred	100262608	60S ribosomal protein L17-2
20	G-green	F-darkred	100262686	40S ribosomal protein S4-3
21	G-green	F-darkred	100265608	RGG repeats nuclear RNA binding protein A-like
22	G-green	F-darkred	100266487	60S ribosomal protein L10a-1
23	G-green	F-darkred	100852874	eukaryotic translation initiation factor 5A-2
24	G-green	F-darkred	109121414	AB hydrolase superfamily protein YfhM-like
