# Supplementary material for: Dual RNA-Seq Reveals Temperature-Mediated Gene Reprogramming and Molecular Crosstalk between Grapevine and Lasiodiplodia theobromae
Source: J Fungi (Basel). 2023 Dec 14;9(12):1197. doi: 10.3390/jof9121197 (PMC10745131; doi:10.3390/jof9121197)
Supplement: Supplementary file 1 [file jof-09-01197-s001.zip › Table S4.pdf]

Supplementary Table 4. List of *L. theobromae* temperature-correlated genes found in correlated modules between grapevine and *L. theobromae*.

	*L. theobromae*_module	Grapevine_module	Gene	Description
1	F-brown	G-pink	g5563	Fungal specific transcription factor domain
2	F-brown	G-pink	g7634	O-methyltransferase
3	F-brown	G-pink	g12383	ABC transporter protein
4	F-brown	G-pink	g2437	Nudix domain protein
5	F-brown	G-pink	g2480	NADH: flavin oxidoreductase NADH oxidase
6	F-brown	G-pink	g7286	Phenylacetyl-ligase
1	F-red	G-pink	g5330	GTP cyclohydrolase I
2	F-red	G-pink	g9404	Glutathione S-transferase
1	F-darkred	G-pink	g6235	NA
2	F-darkred	G-pink	g6412	Coproporphyrinogen III oxidase protein
1	F-red	G-blue	g5330	GTP cyclohydrolase I
2	F-red	G-blue	g9404	Glutathione S-transferase
3	F-red	G-blue	g9410	NA
1	F-brown	G-blue	g11115	Rta1 domain protein
2	F-brown	G-blue	g12383	ABC transporter protein
3	F-brown	G-blue	g2437	Nudix domain protein
4	F-brown	G-blue	g2480	NADH: flavin oxidoreductase NADH oxidase
5	F-brown	G-blue	g7286	Phenylacetyl-ligase
6	F-brown	G-blue	g7634	O-methyltransferase
1	F-red	G-salmon	g10224	AAA family ATPAse
2	F-red	G-salmon	g2681	Polyketide synthase
3	F-red	G-salmon	g2686	Siderophore transporter, RhtX/FptX family
4	F-red	G-salmon	g3098	Cross-pathway control protein 1
5	F-red	G-salmon	g5330	GTP cyclohydrolase i
6	F-red	G-salmon	g9404	Glutathione S-transferase
1	F-brown	G-salmon	g2437	Nudix domain protein
2	F-brown	G-salmon	g5563	Fungal specific transcription factor domain
3	F-brown	G-salmon	g7069	ATPAse AAA+ type core protein
4	F-brown	G-salmon	g7634	Catechol O-methyltransferase
1	F-darkred	G-salmon	g6235	NA
2	F-darkred	G-salmon	g6412	Coproporphyrinogen III oxidase protein
1	F-brown	G-green	g4867	NA

2	F-brown	G-green	g5563	Fungal specific transcription factor domain
1	F-darkred	G-green	g3337	Phosphoglycerate kinase
2	F-darkred	G-green	g6235	NA
3	F-darkred	G-green	g6412	Coproporphyrinogen III oxidase protein
